# Striking structural dynamism and nucleotide sequence variation of the transposon *Galileo* in the genome of *Drosophila mojavensis*

**DOI:** 10.1186/1759-8753-4-6

**Published:** 2013-02-04

**Authors:** Mar Marzo, Xabier Bello, Marta Puig, Xulio Maside, Alfredo Ruiz

**Affiliations:** 1 Departament de Genètica i de Microbiologia, Universitat Autònoma de Barcelona, Bellaterra, Catalunya, 08193, Spain; 2 Departamento de Anatomía Patolóxica e Ciencias Forenses; Grupo de Medicina Xenómica, Centro de Investigación en Medicina Molecular e Enfermedades Crónicas (CIMUS), Universidade de Santiago de Compostela, Galicia, 15782, Spain; 3 Present address: School of Biomedical Sciences, Queen’s Medical Centre, University of Nottingham, Nottingham, NG7 2UH, UK; 4 Present address: Unitat de Genòmica, Institut de Biotecnologia i Biomedicina, Universitat Autònoma de Barcelona, Bellaterra, Catalunya, 08193, Spain

**Keywords:** Transposable element, *Drosophila mojavensis*, Evolution, Terminal inverted repeat, Phylogeny, Genomics

## Abstract

**Background:**

*Galileo* is a transposable element responsible for the generation of three chromosomal inversions in natural populations of *Drosophila buzzatii*. Although the most characteristic feature of *Galileo* is the long internally-repetitive terminal inverted repeats (TIRs), which resemble the *Drosophila Foldback* element, its transposase-coding sequence has led to its classification as a member of the *P-element* superfamily (Class II, subclass 1, TIR order). Furthermore, *Galileo* has a wide distribution in the genus *Drosophila*, since it has been found in 6 of the 12 *Drosophila* sequenced genomes. Among these species, *D. mojavensis*, the one closest to *D. buzzatii*, presented the highest diversity in sequence and structure of *Galileo* elements.

**Results:**

In the present work, we carried out a thorough search and annotation of all the *Galileo* copies present in the *D. mojavensis* sequenced genome. In our set of 170 *Galileo* copies we have detected 5 *Galileo* subfamilies (C, D, E, F, and X) with different structures ranging from nearly complete, to only 2 TIR or solo TIR copies. Finally, we have explored the structural and length variation of the *Galileo* copies that point out the relatively frequent rearrangements within and between *Galileo* elements. Different mechanisms responsible for these rearrangements are discussed.

**Conclusions:**

Although *Galileo* is a transposable element with an ancient history in the *D. mojavensis* genome, our data indicate a recent transpositional activity. Furthermore, the dynamism in sequence and structure, mainly affecting the TIRs, suggests an active exchange of sequences among the copies. This exchange could lead to new subfamilies of the transposon, which could be crucial for the long-term survival of the element in the genome.

## Background

Transposable elements (TE) are genetic entities capable of changing their location in the genome [1]. Because of their disperse and repetitive nature, they are considered part of the middle repetitive DNA portion and they make up significant fractions of different genomes, such as 14% in *Arabidopsis thaliana*, approximately 15% in *Drosophila melanogaster*, approximately 45% in humans and approximately 80% in some crops [2-5]. They have been found in virtually all the studied species, showing a very old origin and a remarkable persistence over evolutionary time [6]. Since their new insertion sites are usually random, they are considered to be mutational agents, which allowed them to be firstly considered as junk DNA [7,8]. Nevertheless, they can be taken as powerful facilitators of evolution, since they generate variability, the raw material for evolution, along with some adaptive TE insertions which have been reported [9,10].

Since TEs present huge variability in length, structure and transposition strategies, a classification system is needed to understand and handle all the information about this type of DNA. Although classification criteria have not reached a complete consensus, there is general agreement about the first split in the classification: the existence or not of a retrotranscription step [11]. Structural and homology criteria are used to further classify the different elements in subclasses, orders, superfamilies and families [4,6,12].

Terminal inverted repeat (TIR) DNA transposons (Class II, subclass I) comprise those elements without the retrotranscription step and with TIRs [4]. These elements are mobilized by a transposase protein encoded by autonomous or canonical copies of the element using a cut-and-paste mechanism. Apart from transcription-active (canonical) copies of a transposon family, most genomes also harbor defective copies which are unable to encode a functional protein and, thus, are non-autonomous. These copies appear due to mutations in the canonical-structured elements, along with genomic deletion and unequal exchange after non-allelic homologous recombination (NAHR) this way, the transposon activity generates deletion derivative copies [13,14]. These defective copies usually present a gradient of random deletions and there are almost-complete copies down to copies that are only made up of TIRs and a spacing region [6,14,15]. Furthermore, there is a special kind of defective element called MITE (Miniature Inverted repeat Transposable Element), which is mainly defined by its very high copy numbers and short length. MITEs can be considered as deletion derivatives, but in some cases, they seem to have acquired non-related sequences and only present homology to the canonical copies in the TIRs or the very ends of the TIRs [16]. These MITEs use or parasitize the transposition machinery coded in the complete copies and have been called the ultimate parasites [17,18].

*Galileo* is a transposable element discovered in *D. buzzatii* where it has been responsible for the generation of three natural chromosomal inversions [19-21]. Because the first copies of *Galileo* were only made up of long TIR sequences, it was tentatively classified as a *Foldback-*like element [22,23]. However, when the *Galileo* transposase sequence was discovered, it was definitely classified as a member of the *P-element* superfamily of DNA transposons (class II, subclass I and TIR elements order), being the longest TIR element (from about 300 bp to 1.2 kb TIR length) of its superfamily [24]. Despite the first studies that pointed out that *Galileo* distribution was limited to the species closest to *D. buzzatii*[23], bioinformatic analysis of the 12 sequenced *Drosophila* genomes uncovered a broader distribution, because 6 of the 12 species harbored it [24]. In this initial bioinformatic analysis, one of these species, *D. mojavensis*, showed a remarkable diversification of *Galileo* sequences, with four phylogenetically differentiated groups and high structural variability among the copies. Both *D. mojavensis* and *D. buzzatii* are members of the *repleta* group of the *Drosophila* subgenus.

In the present work, we carried out a more detailed bioinformatic search and analysis of the transposon *Galileo* in the *D. mojavensis* genome. We identified 170 *Galileo* copies using different automated searching strategies coupled with a detailed manual annotation in each of them. A huge variability in length and structure was found, with sequences ranging from nearly-complete copies to only two TIR elements. In addition, the sequence diversity found allowed the description of five *Galileo* groups/subfamilies, one more than the previous work; four of them harbor defective transposase sequences and one of them could have a chimeric origin. The activity of *Galileo* copies in *D. mojavensis* was explored through Bayesian analysis, and the results suggest that this transposon has been active until recently or maybe it could still be active. Finally, the structural dynamics, which comprise TIR extension, have been analyzed in detail and mechanisms for this dynamism are discussed.

## Results

### *Galileo* searches

**Figure 1:**
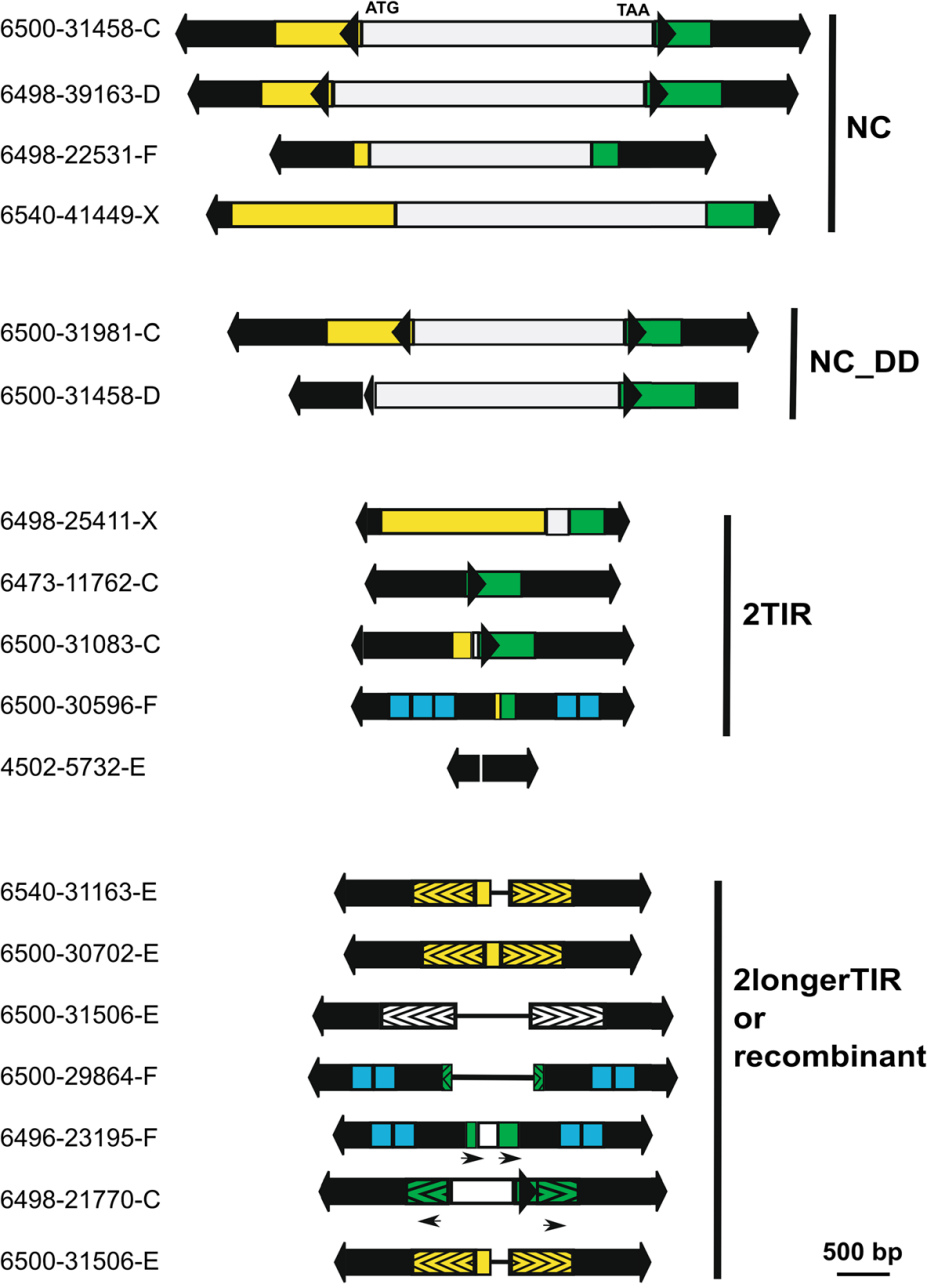
**Structures of representative *Galileo *copies found in the *D. mojavensis *genome.** The black arrows are the TIR; the grey middle region is the transposase sequence; the yellow region is the F1 (spacing sequence between the TIR 1 and transposase coding segment); the green region is the F2 (spacing sequence after the transposase-coding segment and the TIR-2). The blue squares are tandem repeats found in the F group. The region with bracketed pattern (>>>) is the extra TIR region recruited in the extended TIR copies. The black arrowheads are internal short inverted repeats found in C and D groups. NC copies are nearly-complete copies, NC_DD are deletion derivatives of the nearly-complete ones. TIR, terminal inverted repeats.

Different bioinformatic search strategies were used to maximize the probability of finding *Galileo* copies (see Methods). A total of 170 *Galileo* copies were identified and manually annotated (a 370% sample size increase over the 36 previously described copies [24]). These copies were classified according to subfamily, structure and chromosomal distribution (see Table 1 for a summary and Additional file 1: Tables S1 and S2 for detailed information). Subfamily classification was based on the phylogenetic analysis of TIR sequences and resulted in five well-supported groups (C, D, E, F and X). Twelve copies were found to contain sequences belonging to different subfamilies and were considered as chimeric (Table 1). Structural classification produced five groups: nearly-complete (NC), deletion derivatives (DD), two TIR elements (T2), two extended or recombinant TIR elements (2RT) and solo-TIR (Table 1). Some representative copies of these structural groups are depicted in Figure 1.

**Table 1:** **Summary of the *Galileo *copies studied in this work**

**Structural type**	**Subfamily**						**Total**
	**C**	**D**	**E**	**F**	**X**	**Chimeric**	
Nearly complete (>2 kb TPase)	2	5	0	1	1	1	10
Nearly complete deletion derivatives	4	2	0	1	2	0	9
2 TIR	5	0	7	28	3	6	49
2 recombinant TIR	2	2	22	3	4	5	38
Solo-TIR	6	10	19	26	3	0	64
Total	19	19	48	59	13	12	170
Mean TIR % identity between copies	97.1	96.5	93.9	92	92	79	79.5

The different subfamilies and structures are indicated. The average of pairwise identity in each subfamily is shown (percentage calculated from 238 TIR alignment with MEGA 5.1 [25]). *TIR* terminal inverted repeats.

### *Galileo* subfamilies in the *D. mojavensis* genome

**Figure 2:**
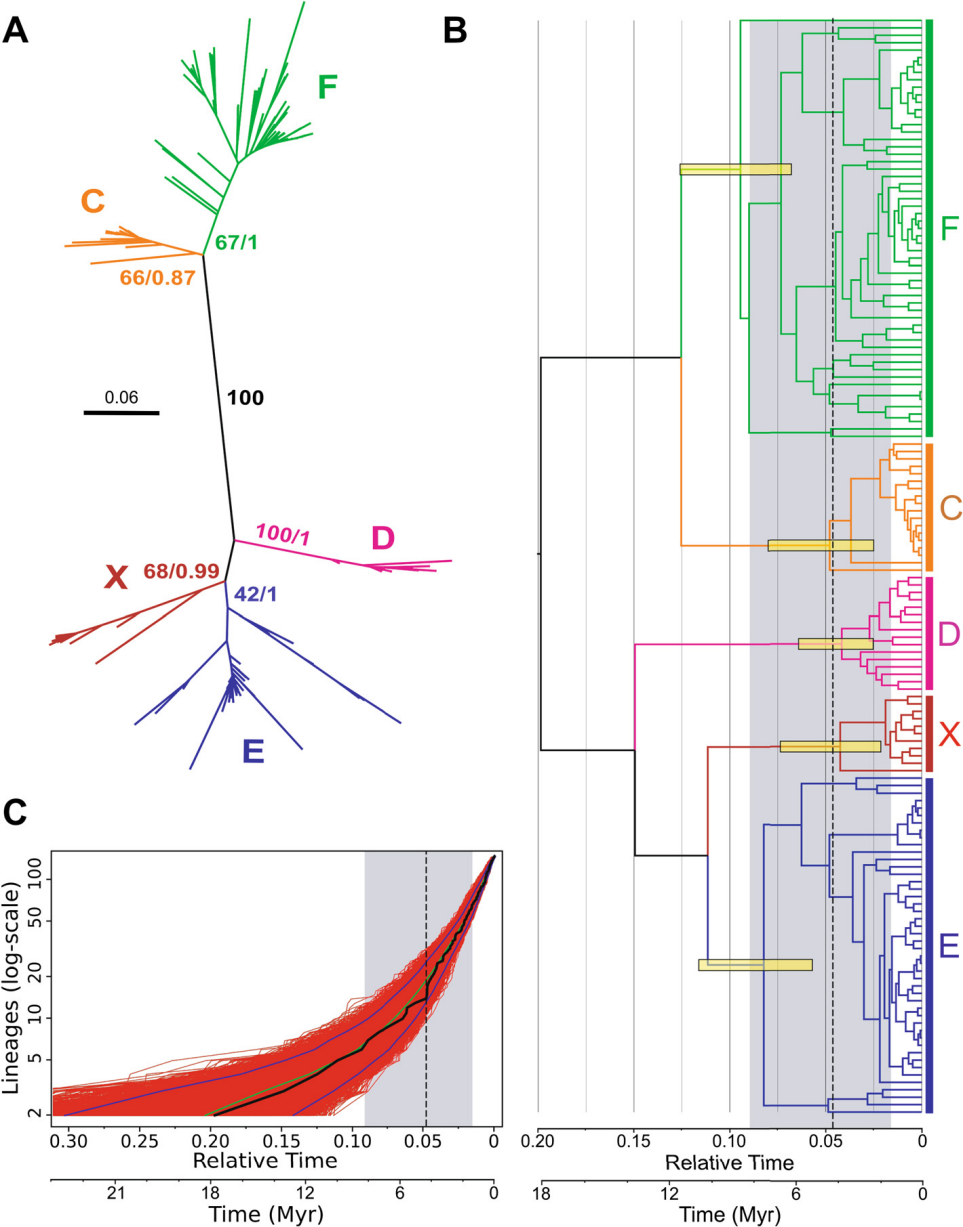
***Galileo *phylogenetic analyses. A**) Unrooted tree inferred using 238 TIR sequences of *Galileo.* Phylogenetic reconstructions were carried out by means of maximum likelihood (ML, PhyML) and Bayesian inference (BI, BEAST) methods using a HKY+G evolutionary model. Numbers on nodes indicate the support of each group as bootstrap and Bayesian posterior probability, respectively. The five groups show strong support. **B**) BEAST ultrametric summary tree inferred using 148 TIR sequences of *Galileo* (only one TIR of each *Galileo* copy was used and chimeric copies were excluded). The yellow bars correspond to the 95% highest posterior density intervals for node ages. The best-fit model of diversification was a yule-2-rate in which a constant duplication rate changes to another constant rate at a certain time, and the discontinuous vertical line indicates the shift in the duplication rate (0.048 substitutions/position, about 4.36 myr) and the grey area represents the 95% confidence interval obtained using 10,000 trees sampled from the Bayesian analysis. **C**) Lineages through time (LTT) plot representing the accumulation of cladogenesis events. Red lines represent the LTT plot for each of the 10,000 trees sampled from the Bayesian analysis. Black and green lines show the median and the mean, respectively. Blue lines represent the 95% credible interval.” This would be followed by the figure abbreviations.

A phylogenetic tree was built using the homologous TIR region of all the copies (Figure 2A). The tree shows five groups with significant statistical support, four of them (C, D, E and F) agree with the previously described *Dmoj\Galileo* subfamilies [24], whereas the fifth, that we have named X, is a novel group (Figure 2A). The general relationship among the groups is similar to that found in the previous work, with two main lineages, one comprises the D, E and X groups, and the other the C and F groups. Furthermore, the phylogeny also detected 12 chimeric copies (not shown in Figure 2A) with the 2 TIR belonging to different phylogenetic groups. In addition, these copies are flanked by non-matching 7 bp sequences instead of identical direct target site duplications (TSD) as most other copies. It could be possible that these chimeric copies are a by-product of the genomic assembly. However, the fact that they are located in long scaffolds of the genome suggests to us that they are located in reliable sequenced genomic regions.

In order to explore the evolutionary dynamics of *Galileo* copies through time, an ultrametric tree was generated using a relaxed molecular clock (Figure 2B). In this case, only one TIR sequence per *Galileo* copy was included (usually TIR1, and in some cases TIR2 when TIR1 was not present or was too short) and chimeric copies were omitted. In this tree we included an estimation of absolute time, which provides ages for each node. If we take into account the common ancestral node for each one of the *Galileo* subfamilies, different ages are found. For example, the last common ancestral node for all the F copies is approximately 8.6 million years (myr), which means this group would be the first one diversifying in this genome. It would be followed by E (approximately 7.45 myr), C (approximately 4.35 myr), D and X (these last two less than 4 myr). Most of the copies (approximately 60%), regardless of the phylogenetic group, seem to be quite recent as they appeared in the last million years. In addition, the cumulative graphic of lineages through time (LTT plot) showed an exponential growth of the number of *Galileo* sequences without any apparent deceleration in the curve (Figure 2C). Thus, *Galileo* has not stopped its transposition activity in the time depicted in the graphic. Furthermore, we have performed a diversification rate test and at least one shift has been detected which is located in 0.048 relative time units (substitutions/position) (about 4.36 myr vertical discontinuous line in the tree, Figure 2B and C) where the rate of *Galileo* proliferation changes from 16.28 sequences/relative time units to 48.66 sequences/relative time units (95% confidence interval for each rate: 5.87 to 30.31 and 39.77 to 58.24 lineages/time). These observations indicate that *Galileo* is still active or has been active until very recently in the *D. mojavensis* genome.

**Figure 3:**
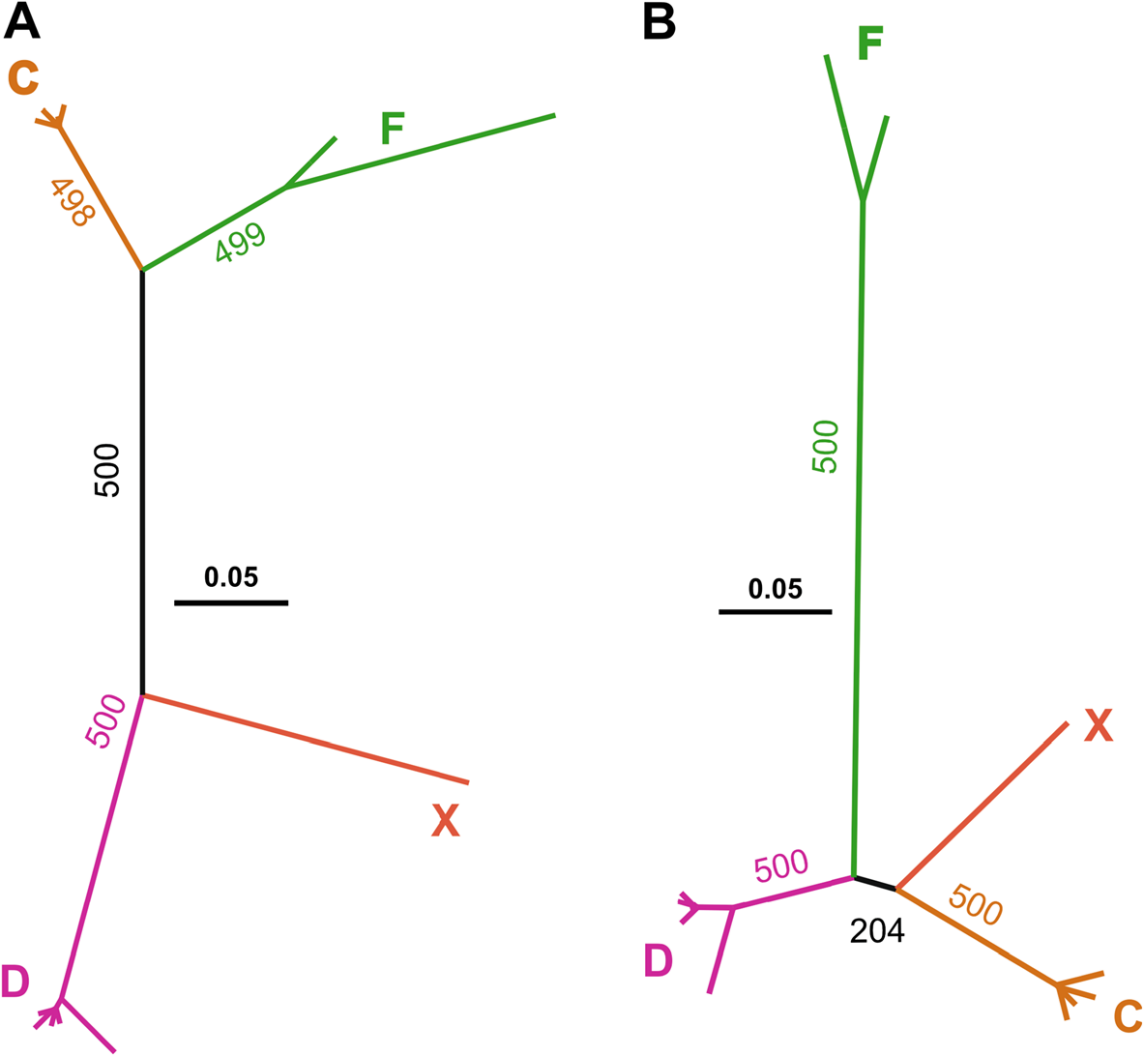
**TIR and transposase coding region phylogenies.** Twelve *Galileo* elements were used for these analyses. **A**) TIR phylogeny. **B**) Transposase phylogeny, PhyML analysis with JC+G+I evolutionary model. The AU test was performed to compare the two tree topologies. AU, approximately unbiased; TIR, terminal inverted repeat.

Twenty *Galileo* copies were found to contain variable portions of the transposase-coding region (Table 1, Additional file 1: Table S1), yet none of them harbors an intact open reading frame (ORF) that can be translated into a functional protein (that is, all of them contain stop codons and/or deletions and frame-shift mutations). These copies belong to subfamilies C, D, F and X, whereas no copies of the E subfamily contain any trace of the transposase-coding region. A phylogenetic tree was built with transposase-coding sequences longer than 2 kb found in the different subfamilies (12 *Galileo* copies in total, see Methods). For comparison, the TIR region of these 12 copies was used to generate a new tree with the same methods. Both phylogenetic trees were similar and recovered the same groups (Figure 3, Additional file 1: Table S3). However, the relationship among the subfamilies seems somewhat discordant: in the transposase-coding region tree groups F and D belong to one of the main lineages, and groups X and C belong to the other, whereas the TIR tree shows the same relationship between groups found previously in the global TIR tree (Figure 3A and B). Differences in topology can be due to different evolutionary histories, but also to phylogenetic uncertainty. In fact, the grouping of F and D in the transposase-coding tree has a low bootstrap support (41%). Moreover, an approximately unbiased (AU) test was performed (CONSEL program) to test if any of the two topologies could be significantly rejected using the information in both alignments. Using this approach, neither of the two topologies could be rejected in the case of the transposase alignment (TIR topology: *P* = 0.39, transposase-coding topology: *P* = 0.61), indicating that information in the alignment does not allow discriminating between both phylogenetic hypotheses. However, when the TIR alignment was used, we found that the transposase-coding topology was significantly rejected (TIR topology *P* = 1; transposase-coding topology *P* = 7e-11). These results suggest that the position of the F subfamily in the transposase coding segment tree might be biased, as a consequence of the reduced number of sequences used, phylogenetic noise in this *Galileo* region or recombination.

### *Galileo* structural variation

*Galileo* copies exhibit a striking amount of structural variation (Figure 1). For the purpose of description and analysis, we have grouped all copies into five structural groups: NC, DD, 2T, 2RT and solo-TIR (see methods). All phylogenetic groups except D and E contained copies of the five different structures described (Table 1). The D subfamily lacked 2T elements, whereas the E subfamily did not contain any copy with transposase sequence (neither NC nor DD).

The *Galileo* TIR, defined as the terminal sequence inverted and repeated in each end, is the most variable region among the copies of the element, not only in nucleotide sequence as phylogeny shows but also in length. TIR length varies from 18 bp to 1,250 bp with a total average of 668 bp. The variation of TIR length is found in all the subfamilies [see Additional file 1: Table S1 where means and standard deviations are given], but when the means of the five subfamilies are compared, the only pairs of comparisons that present statistical differences are between the X and E subfamily and X and F subfamily (Tukey-Kramer means comparison test, *P* <0.05). The X subfamily possesses the shortest TIR and subfamilies E and F the longest TIRs. When the TIR length is compared among the different structural types, the only significant length difference is found between the 2T and the 2RT type, which is in agreement with the classification criterion (Tukey-Kramer means comparison test, *P* <<0.05). We have explored the sequences comprising the TIRs. Generally, the shortest TIRs are due to the lack of TIR sequence in one of the *Galileo* ends. Thus, although one transposon end still possesses a whole TIR, the repeated span gets shorter because of the sequence missing in the other end (it is not repeated any more). This is how some very short TIRs are found in copies such as the F subfamily 6680–244202 or X subfamily 6498–95069, E subfamily 4198–1393 or C subfamily 6540–613211 (see copy 4502-5732E in Figure 1).

On the other hand, when the longest TIRs are explored, we have observed differences among the subfamilies. For example, in the F subfamily, the presence of direct tandem repeats inside the TIR (located approximately 264 to 467 bp from the TIR end) seems to account for part of the variation in the TIR length. There are TIRs with no internal repeats and TIRs with two or three copies of the internal tandem repeat. Since the tandem repeat region is approximately 210 bp long, when three copies of this sequence are present, TIR length increases by approximately 420 bp. This fact was found in the TIR1 of 6500–30596F and 6500–31107F which are 1,264 bp and 1,263 bp long because they harbor three internal tandem repeats. In contrast, copies 6540–32286F or 6540–57500F harbor 892 bp TIRs due to the lack of internal tandem repeats. It is noteworthy that the tandem repeat expansion and contraction was only found in the F group and was located always in the same region of the TIR, except in copy 6500–30494F which harbored two tandem repeats located 196 to 101 bp from the TIR2 terminal end.

In the other groups, although the tandem repeat structure in the TIR was not found, some copies also showed longer TIRs, when compared to the NC copies. In these cases, the detailed exploration of the TIR sequences uncovered the recruitment of non-TIR *Galileo* sequences (usually the region found immediately after the TIR in the NC *Galileo* element) to generate a longer TIR. For example, part of the sequence of the F1 area (the sequence after TIR1 but upstream of the transposase coding segment) appeared repeated in inverted orientation immediately before the beginning of the TIR2 extending the repetitive span inside the *Galileo* element. In this way, an originally non-duplicated nor repetitive *Galileo* sequence made up a longer TIR. We observed that the extra region of TIRs can come both from the F1 or the F2 region; however, the F2 region appeared duplicated only in the groups C (2 copies) and F (once as a direct repeat, another time as an inverted repeat and it is found in a chimeric copy, as well) whereas the F1 region appeared repeated in the C, D (2 copies), E (22 copies) and X (4 copies plus 2 chimeric) groups.

The *Galileo* copy with the longest TIRs showed a combination of the two expansive traits: tandem repeat expansion (two copies of the tandem repeat in each TIR) along with the recruitment of 121 bp of the F2 sequence in the TIR. This copy is 6500–29864F [see Additional file 1: Table S2], and has TIR lengths of 1,260 bp and 1,241 bp (TIR1 and TIR2, respectively with a 95.2% nucleotide identity). The second and third longest TIR copies belonged to the C group, where two 2RT copies recruited the F2 region for the TIR reaching 1,107 bp long. The next longest copy was found in the E group, followed by copies in the D and X groups [see Additional file 1: Table S2]. It is noteworthy that the copies with the longest TIRs were never nearly-complete ones but non-autonomous copies without the transposase-coding ORF, that is, 2T and 2RT copies [see Additional file 1: Table S1 and Additional file 1: Table S2]. All *Galileo* subfamilies present substantial TIR length variation, because in all groups there are copies with very short and very long TIR.

### Chimeric copies

Twelve *Galileo* copies were composed of two TIRs with an unusually high nucleotide divergence and were bounded by different 7-bp sequences instead of identical TSD [see Additional file 1: Table S2] The TIR phylogeny confirmed that these *Galileo* copies were chimeric (not shown). Structurally, one of these copies was NC and all the others are 2T. Regarding the subfamily, there are 4 F/C (including the NC), 1 F/D, 2 E/F, 1 E/C and 4 F/X. The contribution of each subfamily to the chimeric copies is in agreement with its abundance (Chi square test, *P* >0.05). The fact that F TIRs were more frequent in the chimeric copies would be due to the larger number of F copies in the genome. On the other hand, we have tested if the different subfamilies are randomly combined or whether there are subfamily preferences when the chimeric copies are generated. We have not detected any significant departure from randomness (*P* >>0.05).

We have detected the presence of another kind of chimeric copy, with the two TIRs from the same phylogenetic subfamily, but the internal region from another one. Furthermore, the central region of all these copies seems to have the same origin, the central region of 6680-240698D, one of the 2RT copies of the D subfamily. The central region of this copy presents 441 bp of F1 duplicated and inverted expanding the TIR length. When the E subfamily was explored, the central region of its copies presents high identity to this internal region of the 6680-240698D copy (98% identity), while the 570 bp of the end of each TIR presents 77% identity and, as the phylogenies show, belong to different subfamilies. Likewise, we have found this same central region in two 2T copies classified in the X group (copies 6498–29033 and 6500–29395, classified as X group, approximately 1,640 bp total length). Thus, the same central region was found accompanied by TIRs from three different subfamilies: D, E and X.

### *Galileo* chromosomal distribution and relationship with genes

We have analyzed the interchromosomal and intrachromosomal distribution of the *Galileo* copies [see Additional file 1: Tables S3 and S4]. A total of 138 of the 170 *Galileo* copies are located in scaffolds assigned to the *D. mojavensis* chromosomes [26]. The remaining 32 copies are located in scaffolds that are likely to contain pericentromeric heterochromatin and have not been assigned to any chromosome yet. The distribution of the 138 copies was 29, 26, 43, 14, 3 and 23 for the *D. mojavensis* chromosomes X, 2, 3, 4, 5, and 6 (dot), respectively. This interchromosomal distribution shows a significant departure from a random distribution (taking into account chromosome size, chi square test *P* <<0.05). There is an excess of *Galileo* copies in the dot chromosome, whereas fewer than expected copies are found in chromosome 5.

In addition, we have explored the intrachromosomal distribution of *Galileo* copies. In the *D. mojavensis* there are three chromosomes (2, 3 and 4) each represented by a single major scaffold (6540, 6500 and 6680, respectively) [26]). We have subdivided these scaffolds in distal (10% of the sequence), central (80% of the sequence) and proximal (or centromeric, 10% of the sequence) segments in relation to the position of the centromere, and tested if *Galileo* copies present a uniform distribution in these regions. We observed a very significant departure from what was expected by chance, since *Galileo* copies tend to accumulate in the proximal region near the centromere (*P* <<0.01, in the three cases, Additional file 1: Table S4).

Furthermore, coordinates of *Galileo* copies have been compared to those of the predicted genes in the *D. mojavensis* genome (including protein-coding and non-coding RNA genes). The 170 *Galileo* copies were classified as follows: 23 are located in scaffolds without genes, 23 are located inside genes (all of them inside introns) and 124 are located in intergenic regions [see Additional file 1: Tables S5 and S6]. The distance of the intergenic *Galileo* copies to the nearest gene ranged from 29 to 110,537 bp (average 11,439 bp, median 5,253 bp). No correlation was observed between copy length and distance to the nearest gene (Spearman’s rho *P* >>0.05), or between copy length and intergenic region length (Spearman’s rho *P* >>0.05). There was no differential distribution regarding the 5′ or 3′ gene regions (chi-square test *P* >>0.05), neither when the different subfamilies (*P* >>0.05, from 1 to 0.36) nor when the structural *Galileo* type (*P* >>0.05, from 0.22 to 1) were taken into account.

A set of 17 *Galileo* copies is located very close to genes (less than 500 bp, see Additional file 1: Table S5) and 14 of them possess a *D. melanogaster* ortholog. The functions of these genes have been explored and they are involved in different cellular processes, such as tRNAs, methyl transferases, helicases, and DNA binding proteins. Another group of copies (23 *Galileo*) have been found inside genes. In all cases, the *Galileo* elements were located inside 16 different introns (in some introns there were more than one *Galileo* element). The length of these introns ranged from 1,478 to 172,415 bp, and 10 of the 16 genes whose introns harbored *Galileo* copies have been assigned an ortholog gene in *D. melanogaster* [see Additional file 1: Table S6]. There was no correlation between *Galileo* length and intron length; neither type nor subfamily is over-represented inside the genes (*P* >>0.05).

## Discussion

In a previous work, we uncovered the presence of *Galileo* elements in 6 of the 12 sequenced *Drosophila* genomes [24]. Among them, the *D. mojavensis* genome showed the highest variability in *Galileo* sequence and structure. A small sample of 16 nearly-complete copies that contained transposase-coding sequences and 20 non-autonomous copies was analyzed. Analysis of the TIR sequence variation showed that the copies clustered in four different groups or subfamilies (that were named C, D, E and F). Two of these subfamilies, C and D, harbored truncated transposase coding regions, while the other two groups were only composed of non-autonomous copies (mainly 2 TIR structure). The existence of different groups in the same genome suggested several amplification bursts in the past. Furthermore, a high variability in TIR length was detected. Since the TIR length is the most characteristic feature of *Galileo* elements, the *D. mojavensis* genome offered the opportunity to study this trait in detail.

Here, we carried out a thorough analysis of *Galileo* variation and distribution in the *D. mojavensis* genome sequence. In the present work we have uncovered the existence of at least five subfamilies of *Galileo* elements. Four of them contain nearly complete copies with transposase-coding segments, which implies the putative co-existence of four fully functional subgroups. The co-existence of different subgroups or subfamilies has previously been reported for *D. melanogaster P-element* and other transposons [27-30]. There are two main hypotheses that would explain the co-existence of different subfamilies in the same genome: horizontal transfer (HT) and genomic vertical diversification. Under the first hypothesis, in the case of HT events, the *Galileo* element could have arrived to *D. mojavensis* via some close spatiotemporal species, such as mites or other intimate parasites [31-34]. If the five subfamilies (C, D, E, F and X) had arrived through this mechanism, this would imply at least five independent events of successful HT and invasion of the *D. mojavensis* genome. If our estimation of the age of each subfamily is taken into account, these horizontal transfer events would have happened in an approximately 5 myr period, which would mean an average of one horizontal transfer event per myr. When the variability of the age nodes is taken into account, this time range reaches approximately 9.5 myr (from 0.125 to 0.02 changes/time, 11.36 and 1.81 myr, respectively), which would mean approximately 0.53 horizontal transfers per myr. This would imply something like a ‘*Galileo* bombing’ against *D. mojavensis* genome in the past. This HT rate is higher than the 0.04 HT/myr/family obtained by Bartolomé *et al*. [35]; even if we divide our estimation among the number of *Galileo* subfamilies, we still get a higher rate of 0.1 HT/myr/subfamily. This massive HT seems very unlikely.

On the other hand, the different *Galileo* subfamilies could have diverged vertically from an ancestral resident in the genome. This putative ancestor sequence would have existed approximately 18 myr ago (0.20 units/relative time, considering 0.011 changes/position/myr [36]), as seen in our Bayesian ultrametric tree (BEAST) (Figure 2B). Such functional differentiation could have been driven by specific selective pressures to form several subfamilies producing distinct *Galileo* transposases to overcome the cell transposition repression. When a new transposase appears along with high-affinity sequences, a transposition burst would happen. After that, truncated copies of the successfully transposed ones would appear, rendering deletion derivatives, 2T, 2RT and solo-TIR copies. In each subfamily, all these structural types would appear independently and could spread while they conserved the affinity for the enzymes encoded elsewhere in the genome by an autonomous copy [17,18,37]. This is the landscape *Galileo* presents in the *D. mojavensis* genome.

Another factor that could influence the *Galileo* diversification would be the genetic drift, which is very sensitive to the host population structure. *D. mojavensis* is a species with very divergent populations that are considered as geographical races or even subspecies. It could be possible that a different *Galileo* subfamily evolved in each isolated population and secondary contacts between these populations mixed the different groups. However, our time estimation of each subfamily is not in agreement with the putative ages of the different *D. mojavensis* races, which would probably be less than one myr [38,39]. Thus, population structure seems not to explain the existence of *Galileo* subfamilies in *D. mojavensis*.

Nevertheless, the two explanations, horizontal transfer and genomic vertical diversification, are not mutually exclusive. Thus, a combination of the two phenomena could have happened. However, vertical diversification of *Galileo* subfamilies seems at this time more parsimonious. Our estimations indicate that the *D. mojavensis Galileo* subfamilies had a common ancestor approximately 18 myr ago. This is showing us that *Galileo* has an old history in *D. mojavensis*, which is in agreement with the *Galileo* ancient origin in the genus [24]. Likewise, recent data have uncovered the existence of *Galileo* elements in many other members of the *Drosophila repleta* species group, besides *D. buzzatii* and *D. mojavensis* (Andrea Acurio, Deodoro Oliveira and Alfredo Ruiz, in preparation). However, although the *Galileo* last common ancestor in the genus could be as old as the origin of the *Drosophila* genus, the subfamilies found in *D. mojavensis* diversified quite recently (4 to 9 myr ago). Consequently, only closely related species to *D. mojavensis* are expected to harbor these very same subfamilies, and different subfamilies probably exist in more distantly related species.

The genomic dynamics of transposons helps us to understand the variety of *Galileo* copies in the *D. mojavensis* genome. The natural cycle of a DNA transposon would begin with the invasion of a new genome by a fully functional transposon, through horizontal transfer [32,34,37] or perhaps by remodeling/reactivation of an inactive one. After that, since class II transposition depends entirely on the cell replication and repairing machinery of the double-strand breaks (DSB), the truncated copies start to appear due to errors in the repair process. Likewise, the truncated copies that would maintain the sequences recognized by the transposase, would be able to spread better than the complete copies, probably due to overcoming the putative length penalty some transposons suffer [40]. Moreover, even shorter copies would appear, the so-called MITEs and, eventually, the transposon would become inactivated and disappear [6,32].

*Galileo* element structures clearly show this dynamic. The nearly-complete copies are 5.2 kb average length and a gradient of shorter copies with different deletions appeared. This way, there is a group of copies where no transposase sequence is found and they are composed almost entirely of TIR. Maybe these copies could be considered as *Galileo* MITEs but there are some drawbacks for this definition. First of all, the main trait of a MITE is its length, usually less than 600 bp [4,6,41]. *Galileo* 2-TIR elements are 1.7 to 2.2 kb average length, mainly due to the TIR length *per se*. Secondly, although the 2TIR copies outnumber the nearly-complete ones, the number of copies is not as high as the thousands of copies reached by MITEs in some genomes [6]. Finally, since in *Galileo* the changes from the most complete copies to the 2TIR elements are traceable virtually in all copies, we propose a 2TIR-element tag for this deletion-derivative kind of *Galileo* copies.

Regarding the *Galileo* TIR dynamics, we have observed length expansion and contraction. On the one hand, for the contraction, the genomic deletion rate in TEs has been studied and would explain how this would happen [13]. On the other hand, the expansion of the TIR would be a bit more complex than deletion. The expansion of the TIR in the F groups is mainly due to the expansion and contraction of the direct tandem repeats which are located inside the TIR. A different number of tandem repeats are found when the two TIRs of a *Galileo* F copy are compared, rendering independent TIR dynamism. This would be in agreement with the statement that any region generated by duplication can thereafter be duplicated [42,43]. Furthermore, the tandem repeats in the TIR or in subterminal regions of transposons have been proposed to harbor secondary binding sites for the transposase [30,44-46]. In our case, *Galileo* elements also present these tandem repeats (subfamilies G and F [23,24]) and they contain secondary binding sites at least in *Dbuz\GalileoG* (Marzo M, Liu D, Ruiz A and Chalmers R, submitted). The multiple binding sites seem to be a convergent trait that appears in different transposable element superfamilies and could be positively selected for an improved transposition reaction, thanks to a higher transposition machinery affinity.

Besides the tandem repeat expansion, we have detected another source of TIR extension: the recruitment of internal sequences to extend the TIR. This could be due to the structure of the *Galileo* sequences, where two close inverted repeats of at least 600 bp long might attract recombination, whether due to the DSB after transposon excision, the structural instability or ectopic recombination as a result of being a genomic dispersed repetition. We could suggest that *Galileo* would behave similarly to the segmental duplications in addition to its transpositional nature. Segmental duplications are repetitive regions of the genome that are able to recombine, exchange and convert sequences [47]. For example, if a *Galileo* copy suffers a DSB in the TIR2 (due to a problem during the replication step, for example) it could be repaired through NAHR. If for repairing this TIR2, it uses as template the TIR1 of a copy of the same subfamily (the two TIR present 98% to 100% nucleotide identity between the TIRs of the same *Galileo* copy), the copied tract could be longer than the TIR1 and include other internal regions of the element. In that case, since the TIR1 is being copied where the TIR2 is located, the region that was downstream of the TIR1 would appear upstream of the TIR2 as well, becoming a repetitive sequence in inverted orientation and extending the TIR span. The result is TIR1-F1-F1-TIR2. The expansion of inverted repeat sequences has been reported for segmental duplications and *Polintons* inverted repeats (TE); thus, the dynamics of inverted repeats seems a general genomic dynamic trait [12,43,48].

Then, we can imagine that ectopic recombination and genomic conversion would be acting among all *Galileo* copies and different products could appear, among them the chimeric elements. In these cases, if one of the exchange breakpoints (of the conversion tract) is located inside the element, it would generate a chimeric element with two well-defined segments from two different subfamilies. These chimeric copies resemble the *Galileo* copies found in the breakpoints of polymorphic inversions in *D. buzzatii* which is in agreement with the *Galileo* inversion generations due to ectopic recombination [19-21]. Furthermore, if the two exchange breakpoints are located inside the element, this would produce, for example, the X-E-X copies and, probably, this could be the origin of the whole E subfamily as well.

We would like to propose that long TIRs, although they imply a handicap for the transposition reaction [40], could be useful for the survival of the transposon: the more the recombination rate among these sequences is due to the length of the TIRs, the more chance there is for a new *Galileo* subfamily to appear. There would be more raw material for the transposase to choose from and a new transposition burst would be triggered. The TIR length dynamics, along with the chimeric origin observed among *Galileo* copies is in agreement with an important dynamic DNA exchange of sequences and recombination [43,47,48]. Thus, this would explain why different non-related class II transposons present subfamilies with long TIRs and why TIR length is not a reliable feature for transposon classification [30,44,46,49].

Generally, the mutations or inactivation of the transposase sequence drives the death of a transposon, because without the transposition reaction there is no duplication of the sequences. The fact that we have not found any *Galileo* functional transposase, points out that *Galileo* may be an inactive element. However, our *Galileo* sequences LTT plot, where the accumulation of nodes in the tree is depicted, did not show any decrease or stationary rate of *Galileo* sequences duplication. Thus, if *Galileo* is not still active, it has stopped working quite recently. In this regard, it is worth mentioning that in genome sequencing projects, there are heterochromatic regions that have not been sequenced. Furthermore, there is much variability among the individuals of a species that is not represented by only one genome sequence. We cannot discard the existence of *Galileo* active sequences in other individuals or other genomic regions of *D. mojavensis*.

## Conclusions

*Galileo* is the long-TIR member of the *P-element* superfamily of class II TEs. Our searches and thorough annotation of 170 *Galileo* copies in the *D. mojavensis* genome has uncovered a huge variability in length and structure. Phylogenetically, the subfamilies clustered together for both TIR and transposase sequences, but the transposase region presented less information to resolve the tree topology of the subfamilies. Furthermore, our LTT analysis showed an exponential growth of the number of *Galileo* sequences without any apparent deceleration in the curve, meaning it may still be active. Regarding the structure of the *Galileo* copies, the striking dynamism principally affects the TIRs. Deletion shortens them, but tandem direct repeats dynamics and new TIR sequence recruitment expands them. We propose that long TIR may attract recombination and conversion. This sequence exchange may enhance the birth of new subfamilies and could explain why long TIR is a convergent trait in different transposon superfamilies.

## Methods

### Bioinformatic searches of *Galileo* copies in the *D. mojavensis* genome

Consensus TIR sequences of previously described Dmoj\*Galileo* subfamilies plus 50 bp overall consensus TIR end were used as query sequences against the CAF1 scaffold assembly of the *D. mojavensis* genome [50]. The searches were carried out using an automated process based on wuBlast (http://blast.advbiocomp.com) and the Chao algorithm [51] for the handling of the sequence discontinuities in the blast searches. The hits were selected using an 80–80 criteria with the query TIR (80% identity and 80% of the length [4]) and were considered as part of the same *Galileo* copy if arranged in the proper orientation at a distance <10 Kb. If one TIR did not meet all the mentioned criteria the 3 kb flanking region where the other TIR would be expected to be found was further explored by blast. More *Galileo* copies were found in this way. When no partner was found for a given TIR in the surrounding area, it was considered as a solo-TIR copy for further analysis.

All hits from each search were manually curated and thoroughly analyzed to discard wrong automated identifications. Decisions on the acceptance of a search hit were based on a comparison with previously characterized copies and the identification of characteristic structures by careful annotation. In this way, we identified the different regions in each *Galileo* copy: the TIRs, the transposase-coding region, and the spacing sequences upstream and downstream of the transposase-coding region (that we have named F1 and F2, respectively). Only sequences showing a clear sign of some of these structures were selected for further analysis.

### Annotation of *Galileo* copies

All selected sequences were manually analyzed and annotated using several tools found in the Geneious 5.1.7 software package. The closest annotated sequence for each new copy was detected by a search with blastn [52] and used as reference for the detailed annotation of the new copy. When a region of a new copy was not located in the chosen reference copy, this region was used as a blast query against different *Galileo* sequences and other *Drosophila* TEs in order to detect regions in common with other *Galileo* copies or TE insertions. The TIR span was determined by aligning each copy with the corresponding reverse complement sequence. All copies were classified by structure in one of the following five categories: i) nearly-complete (NC), when two TIR and more than 2 kb of transposase-coding sequence were found; ii) deletion derivatives (DD), when either two TIR and less than 2 kb of transposase-coding sequence were found, or a complete or partial transposase-coding sequence was found, but only one TIR was identified; iii) two TIR elements (2T), when two TIR separated by a short middle region (usually not coding for transposase) were found; iv) two extended or recombinant TIR (2RT), when two TIR were found and they were either longer than the NC copies or presented duplicated sequences (there had been extra sequence recruited in a longer TIR); and v) solo-TIR, when only one TIR was found. Detailed information of the genome location and annotation of each *Galileo* copy is provided in Supplemental Additional file 1: Table S2.

### TIR phylogeny

A consensus TIR sequence was generated for each putative subfamily and a region of 630 bp at the end of the TIR was delimited as homologous among the different consensus TIRs. This homologous region was located in each TIR from each *Galileo* copy and analyzed further. Homologous TIR regions shorter than 450 bp were excluded from the analysis because the quality of the alignment was affected. In this way, a set of 238 TIRs was generated. These TIR regions were aligned with MAFFT using the following parameters: E-ins-I; –op 1.53; –maxiterate 1000; –genafpair; –ep 0; –inputorder; –kimura 200, as is set in Geneious software [53]. The alignment was filtered with Gblocks 0.91b to remove regions too divergent and poorly aligned [54,55]. Gblocks was set up with relaxed parameter values (Minimum Number Of Sequences For A Conserved Position: 120; Minimum Number Of Sequences For A Flanking Position: 120; Maximum Number Of Contiguous Nonconserved Positions: 10; Minimum Length Of A Block: 5; Allowed Gap Positions: With Half) selecting 53% of the original alignment (547 bp of the 1,018 original positions). JModeltest 1.0 [56] was used to find the substitution model that best fits the data by means of the Akaike Information Criterion (AIC), which was HKY+G (Hasegawa, Kishino and Yano plus gamma [57]). An ML search was performed with PhyML 3.0 (20110304) [58,59] using the Subtree Pruning and Regrafting (SPR) algorithm. The substitution model parameters were estimated by the program, using four categories for the gamma distribution and the statistical support was calculated with 100 bootstrap replicates. Bayesian inference (BI) was carried out with BEAST 1.6.1 [60], using an uncorrelated lognormal relaxed clock (UCLN [61]) and the substitution model from jModeltest. We used a birth-death process as a tree prior setting a uniform (0, 1000) distribution for growth and death rates. All other priors were left with default values. Two Markov chain Monte Carlo (MCMC) runs of 50 million generations were carried out and combined with the LogCombiner program included in the BEAST package. In both cases, the chains were sampled every 1,000 steps and the first 10% of the samples was removed as burn-in. Convergence was ensured by checking that the effective sampling size (ESS) values for all parameters were over 200. We obtained the maximum clade credibility summary tree with median node heights using TreeAnnotator (also included in BEAST package).

### Recent transposition activity

A BEAST phylogenetic inference was carried out with the aim of displaying the relative age of each *Galileo* copy. For this purpose only one TIR region (of at least 450 bp long) was selected from each copy (only one TIR per *Galileo* copy) and chimeric elements were excluded. The BEAST priors were set up as mentioned above with the same evolutionary model (HKY+G). Absolute time estimation was performed using the 0.011 changes/base/myr proposed as the neutral mutation rate in *Drosophila*[36]. After that, a LTT plot was generated which depicts copy accumulation through time [62]. We performed statistical tests to determine the best fitting model to a sample of 9,000 trees from the BEAST inference. The diversification models tested were: pure-birth (constant rate), birth-and-death (constant rate), DDX (variable rate), DDL (progressive change with saturation) and Yule-2-rate (abrupt change of the rate in one point). These models were adjusted by ML and the best one was chosen using AIC (LASER R package). In addition, simulations to test if the best fitting model was due to incomplete sampling or data variability were carried out.

### Transposase-coding region phylogeny

Transposase-coding sequences found in the different groups longer than 2 kb (12 elements: 6498–22531F, 6500–31458D, 6541–16442D, 6540–11758D, 6540–23860D, 6485–39163D, 6540–41449X, 6262–30856C, 6541–11419F/C, 6500–31288C, 6482–60893F, 6262–13889C) were aligned with MAFFT (same parameters as above), and the jModelTest was run to find the best evolutionary model for the transposase-coding sequences. ML and BEAST tree were inferred for these sequences (evolutionary model JC+G+I). The cognate TIR of each copy with a transposase-coding segment >2 kb were aligned with MAFFT and new phylogenies with PhyML and BEAST were obtained. The topologies of the transposase-coding sequences and TIR phylogenies were compared and the differences were evaluated with an AU test performed with the CONSEL program [63,64].

### Chromosomal distribution of *Galileo* copies and relation to protein-coding and RNA genes

The genomic and cytological location of *Galileo* copies was inferred from the scaffold coordinates and the correspondence of scaffolds with polytene chromosomes [26]. In order to analyze the intrachromosomal distribution of *Galileo* copies, each chromosome was divided into three regions: telomeric, central and centromeric, containing 10%, 80% and 10% of the sequence, respectively [23,65]. This was only possible for chromosomes 2, 3, and 4, each of which is represented by a single major scaffold [26]. Statistical analyses of chromosomal distribution were carried out with JMP 8.0.2 (SAS Institute Inc. 2009). The *D. mojavensis* gene annotations were downloaded from Flybase.org (ftp://ftp.flybase.net/releases/FB2011_04/). The coordinates of protein-coding and RNA genes were compared with those of *Galileo* copies using *ad hoc* perl scripts. All *Galileo* copies were classified as located in scaffolds without genes, in intergenic regions or in intronic regions. Statistical tests to compare the total length and TIR length with gene distances were performed with JMP 8.0.2 (SAS Institute Inc. 2009). Information about the gene function was extracted from FlyBase.

## Abbreviations

AIC: Akaike information criterion; AU: Approximately unbiased; bp: Base pair; BEAST: Bayesian evolutionary analysis by sampling tree; BI: Bayesian inference; DD: Deletion derivative; DSB: Double-strand break; HT: Horizontal transfer; LTT: Lineage through time; MITE: Miniature inverted repeat transposable element; ML: Maximum likelihood; NAHR: Non-allelic homologous recombination; NC: Nearly complete; ORF: Open reading frame; TE: Transposable element; TIR: Terminal inverted repeat; TSD: Target site duplication; TPase: Transposase; 2RT: Two recombinant TIR elements; 2T: Two TIR elements.

## Competing interests

The authors declare that they have no competing interests.

## Authors’ contributions

AR conceived the research. MM and XB designed the automated searches. MM annotated and analyzed the data. MP searched for transposase-containing copies. MM wrote the manuscript. AR, XM and XB revised and improved the manuscript. All authors read and approved the final manuscript.

## Supplementary Material

Additional file 1 Table S1 Total element length and TIR length in the different *Galileo* subfamilies and subgroups. **Table S2.** Coordinates and annotations list of the *Galileo* copies analyzed in this work. **Table S3.** Chromosomal distribution of *Galileo* copies in *D. mojavensis*. **Table S4.** Intrachromosomal distribution of *Galileo* elements. **Table S5.** Nearest genes to each *Galileo* copies. **Table S6.** Intronic *Galileo* copies. Click here for file
