# Supplementary material for: Striking structural dynamism and nucleotide sequence variation of the transposon Galileo in the genome of Drosophila mojavensis
Source: Mob DNA. 2013 Feb 4;4:6. doi: 10.1186/1759-8753-4-6 (PMC3573991; doi:10.1186/1759-8753-4-6)
Supplement: Additional file 1 Table S1 — Total element length and TIR length in the different Galileo subfamilies and subgroups. Table S2. Coordinates and annotations list of the Galileo copies analyzed in this work. Table S3. Chromosomal distribution of Galileo copies in D. mojavensis. Table S4. Intrachromosomal distribution of Galileo elements. Table S5. Nearest genes to each Galileo copies. Table S6. Intronic Galileo copies. [file 1759-8753-4-6-S1.pdf]

Table S**1**. Copy total element length and TIR length in the different *Galileo* subfamilies and subgroups.

C

		Total length			TIR length	
	N	Mean	Std. Dev.	N	Mean	Std. Dev.
Nearly complete (>2 kb TPase)	2	5912.5	108.19	2	704.5	82.73
Nearly complete deletion derivatives	4	4070	1185.41	3	730.67	34.00
2 TIR	5	1383.8	530.50	5	318.3	242.31
2 TIR longer	2	3119	0	2	1107	0
solo TIR	6	772.5	171.47	-	-	-
Total	19	2504.5			617.208	

D

		Total length			TIR length	
	N	Mean	Std. Dev.	N	Mean	Std. Dev.
Nearly complete (>2 kb TPase)	5	5283.8	657.41	5	545.2	41.482
Nearly complete deletion derivatives	2	3286	147.08	0	0	0
2 TIR		0	0	0	0	0
2 TIR longer	2	1860.5	443.36	2	735.5	392.44
solo TIR	10	552.2	146.68	-	-	-
Total	19	2222.67			599.57	

E

		Total length			TIR length	
	N	Mean	Std. Dev.	N	Mean	Std. Dev.
Nearly complete (>2 kb TPase)	0	0	0	0	0	0
Nearly complete deletion derivatives	0	0	0	0	0	0
2 TIR	7	1424.86	695.49	7	289.07	225.93
2 TIR longer	22	2114.045	369.76	22	907.21	210.37
solo TIR	19	778.90	285.43	-	-	-
Total	48	1469.29			758	

F

		Total length			TIR length	
	N	Mean	Std. Dev.	N	Mean	Std. Dev.
Nearly complete (>2 kb TPase)	1	0	0	1	733	0
Nearly complete deletion derivatives	1	0	0	0	0	0
2 TIR	28	1424.86	695.49	28	709.88	308.85
2 TIR longer	3	2114.046	369.76	3	1086.83	180.03
solo TIR	26	778.90	285.43	-	-	-
Total	59	1528.42			776	

X

		Total length			TIR length	
	N	Mean	Std. Dev.	N	Mean	Std. Dev.
Nearly complete (>2 kb TPase)	1	5047	0	1	147.5	0
Nearly complete deletion derivatives	2	2249.5	245.37	2	168	0
2 TIR	3	1262.33	666.27	3	311.67	192.84
2 TIR longer	4	1723.25	77.66	4	581.75	28.01
solo TIR	3	517	209.45	-	-	-
Total	13	1675.15			374.55	

Chimeric

		Total length			TIR length	
	N	Mean	Std. Dev.	N	Mean	Std. Dev.
Nearly complete (>2 kb TPase)	1	6239	0	1	873.5	0
Nearly complete deletion derivatives	0	0	0	0	0	0
2 TIR	6	1769.17	389.7474	6	599.67	196.51
2 TIR longer	5	1903.6	576.99	5	491.3	252.22
solo TIR	-	-	-	-	-	-
Total	12	2197.67			528.65	

Total

		Total length			TIR length	
	N	Mean	Std. Dev.	N	Mean	Std. Dev.
Nearly complete (>2 kb TPase)	10	5356.6	745.61	10	588.9	196.24
Nearly complete deletion derivatives	9	3436.11	1047.91	5	505.6	309.12
2 TIR	49	1738.88	562.31	49	571.93	322.95
2 TIR longer	38	2139.5	497.62	38	833.88	271.38
solo TIR	64	741.47	234.82	0	0	0
	170	1755.59	1259.58	102	667.93	317.045

Statistical Tests

1. Total *Galileo* length.

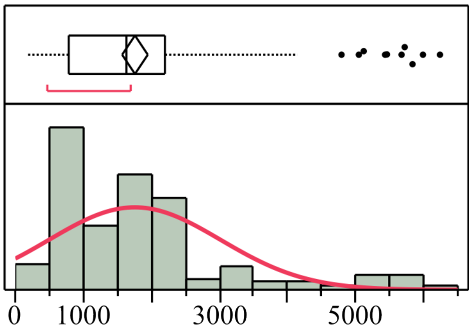

Galileo length distribution

Fitted Normal

Parameter Estimates

Type	Parameter	Estimate	Lower 95%	Upper 95%
Location	μ	1755.5941	1564.8851	1946.3031
Dispersion	σ	1259.5819	1138.4166	1409.8387

-2log(Likelihood) = 2908.54105324984

Goodness-of-Fit Test: Shapiro-Wilk W Test

W	Prob<W
0.834216	<.0001*

Ho = The data is from the Normal distribution. Small p-values reject Ho.

Galileo length by *Galileo* subfamily

Means Comparisons: Comparisons for all pairs using Tukey-Kramer HSD

Abs(Dif)-LSD

	C	D	Chimeric	X	F	E
C	-1148.26	-955.473	-1087.05	-533.405	-58.4314	-28.685
D	-955.473	-1148.26	-1279.84	-726.195	-251.221	-221.474
Chimeric	-1087.05	-1279.84	-1444.87	-894.295	-463.593	-429.642
X	-533.405	-726.195	-894.295	-1388.18	-949.693	-916.45
F	-58.4314	-251.221	-463.593	-949.693	-651.617	-632.487
E	-28.685	-221.474	-429.642	-916.45	-632.487	-722.433

Positive values show pairs of means that are significantly different.

Level		Mean
C	A	2415.6316
D	A	2222.8421
Z.Chimeric	A	2197.6667
X	A	1675.1538
F	A	1540.4915
E	A	1485.0417

Levels not connected by same letter are significantly different.

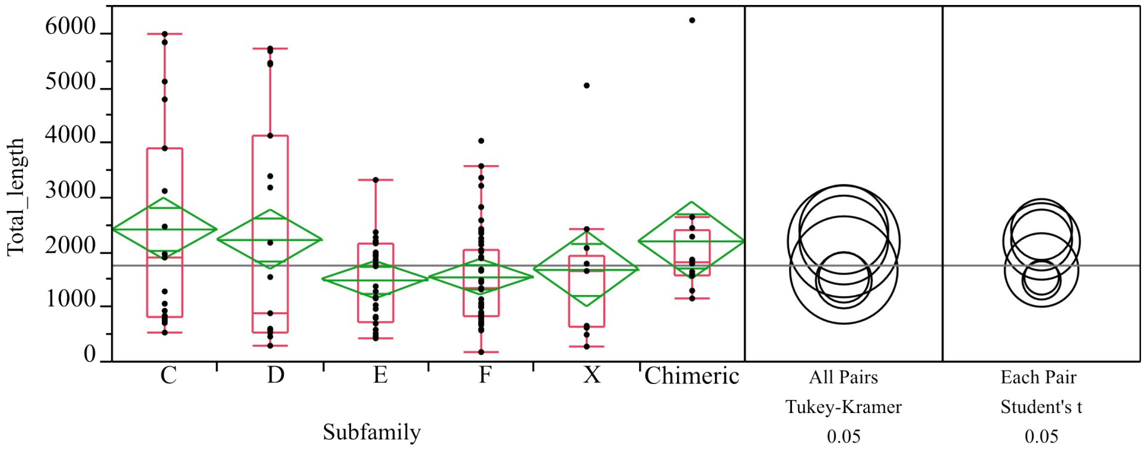

Total length distribution in the different *Galileo* subfamilies of *D. mojavensis*.

Galileo length by *Galileo* structural type

Means Comparisons: Comparisons for all pairs using Tukey-Kramer HSD

Abs(Dif)-LSD

	1.NC	2.NC_DD	4.Longer_2TIR	3.2TIR	5.SOLO
1.NC	-619.371	1284.146	2724.874	3137.145	4144.195
2.NC_DD	1284.146	-652.874	783.1924	1194.971	2201.598
4.Longer_2TIR	2724.874	783.1924	-317.731	101.2543	1114.4
3.2TIR	3137.145	1194.971	101.2543	-279.803	734.511
5.SOLO	4144.195	2201.598	1114.4	734.511	-244.828

Positive values show pairs of means that are significantly different.

Level		Mean
1.NC	A	5356.6
2.NC_DD	B	3436.1111
4.2RT	C	2139.5
3.2T	D	1738.8776
5.SOLO	E	741.4688

Levels not connected by same letter are significantly different.

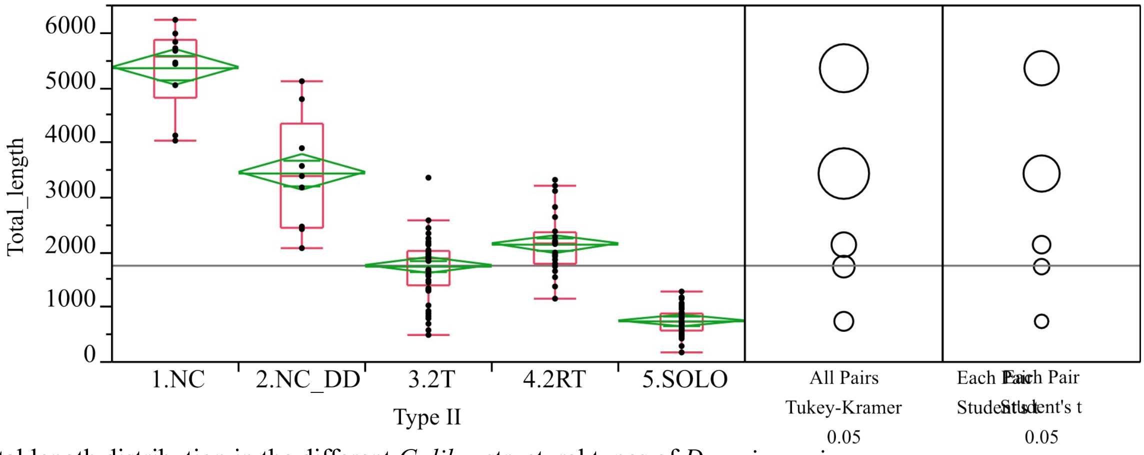

Total length distribution in the different *Galileo* structural types of *D. mojavensis*.

1. *Galileo* TIR length.

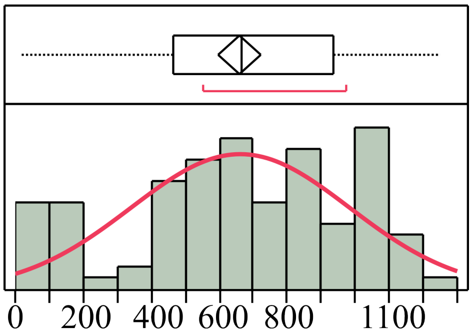

TIR length distribution

Fitted Normal

Parameter Estimates

Type	Parameter	Estimate	Lower 95%	Upper 95%
Location	μ	661.95146	599.16181	724.74111
Dispersion	σ	321.27395	282.58952	372.32654

-2log(Likelihood) = 2908.54105324984

Goodness-of-Fit Test: Shapiro-Wilk W Test

W	Prob<W
0.954506	0.0014*

Ho = The data is from the Normal distribution. Small p-values reject Ho.

TIR length by *Galileo* subfamily

Means Comparisons: Comparisons for all pairs using Tukey-Kramer HSD

Abs(Dif)-LSD

	F	E	C	D	Z.Chimeric	X
F	-223.629	-215.854	-174.065	-226.88	-134.19	47.31895
E	-215.854	-230.963	-188.276	-240.578	-148.401	33.28627
C	-174.065	-188.276	-365.184	-407.789	-325.309	-140.35
D	-226.88	-240.578	-407.789	-478.138	-403.188	-215.8
Z.Chimeric	-134.19	-148.401	-325.309	-403.188	-365.184	-180.225
X	47.31895	33.28627	-140.35	-215.8	-180.225	-400.039

Level		Mean
F	A	745.9375
E	A	734.46667
C	AB	617.20833
D	AB	599.57143
Z.Chimeric	AB	577.33333
X	B	374.55

Levels not connected by same letter are significantly different.

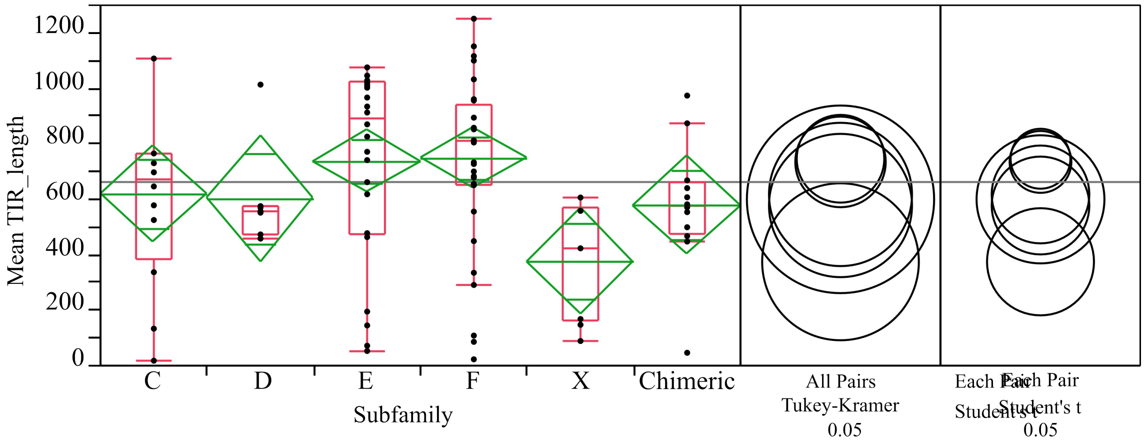

TIR length by Structural Type

Means Comparisons: Comparisons for all pairs using Tukey-Kramer HSD

Abs(Dif)-LSD

	4.2RT	1.NC	3.2T	2.NC_DD
4.2RT	-176.177	-27.9503	95.9579	-37.0464
1.NC	-27.9503	-343.432	-249.502	-337.316
3.2T	95.9579	-249.502	-155.147	-294.2
2.NC_DD	-37.0464	-337.316	-294.2	-485.686

Positive values show pairs of means that are significantly different.

Level		Mean
4.2RT	A	833.88158
1.NC	AB	588.9
3.2T	B	571.92857
2.NC_DD	AB	505.6

Levels not connected by same letter are significantly different.

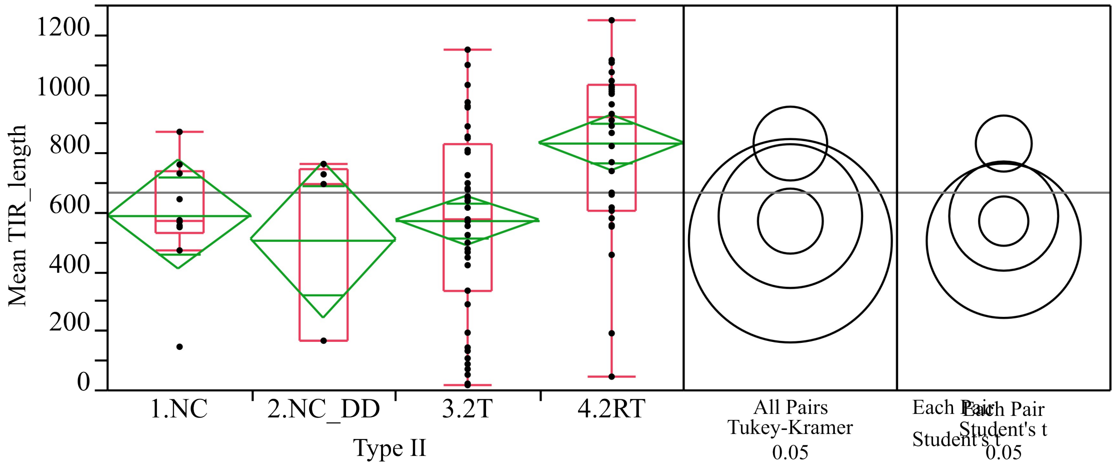

Table S**2**. *Galileo* copies

I. Nearly Complete copies

TAG	Scaffold	Start	End	Subfamily	TSD1	TSD2	Contig	Start	End	Total_length	TIR1 length	TIR2 length	TIR identity	TPase
6500-30856C	6500	30856283	30862823	C	GTTACCG	GTTACCG	10758	37586	44126	5989	813	713	98.7	2784
6500-31288C	6500	31288762	31295303	C	ATGGAGA	TATTGAC	10770	9949	16490	5836	583	709	61.9	2828
6541-11419q	6541	1141978	1149130	Chimeric F/C	GTAGAAC	GTATGGT	11233	4001	11153	6239	788	959	80.1	2808
6485-39163D	6485	39163	45738	D	GTCCAAG	ATTTAAG	9930	1467	8042	5675	574	576	99.3	2814
6498-23860D	6498	2386095	2392524	D	TAATAAA	TAATAAA	10376	4316	10745	5721	570	570	100	2785
6500-31458D	6500	31458921	31465167	D	-	TTTATAT	10773	33627	39873	4130	570	376	94.1	2228
6540-11758D	6540	1175880	1182997	D	CTGAATC	CTAAATC	10946	6739	13856	5433	525	578	89.3	2553
6541-16442D	6541	1644296	1649755	D	AATGTAT	AATGTAT	11255	1328	6768	5460	557	556	99.1	2550
6498-22531F	6498	2253149	2269701	F	CCTGAAC	GTAGCAG	10369	31739	35574	4036	693	773	95.4	2047
6540-41449X	6540	414493	419539	X	-	CTTAAAT	10924	25932	30978	5047	127	168	98.4	2822

II. Nearly Complete Deletion Derivatives copies

TAG	Scaffold	Start	End	Subfamily	TSD1	TSD2	Contig_1	Start	End	Total_length	TIR1 length	TIR2 length	TIR identity	Tpase
6262-13889C	6262	13889	19752	C	no	no	7794	13889	19752	3899	526	0	0	2698
6358-1C	6358	1	5345	C	no	GTACAAT	8435<br>8436	1<br>1	4274<br>736	4793	732	662	99.4	1949
6500-31981C	6500	31981325	31986443	C	AATATAT	AATATAT	10792	22486	27604	5119	815	645	91.9	1951
6482-61400D	6482	614003	617184	D	no	no	9847	20748	23929	3182	254	0	0	1739
6482-61718D	6482	617185	621442	D	no	no	9847	23930	25156	3390	0	0	0	2704
6482-60893F	6482	608936	612509	F	GCGCTAT	no	9847	15681	19245	3574	911	0	0	2323
6406-4469X	6406	4469	6544	X	GCCTTAG	GCCTTAG	8836	146	2221	2076	168	168	97.6	284
6498-25411X	6498	2541172	2544793	X	CTTGTAC	CTTGTAC	10383	26876	30497	2423	168	168	99.4	276

Table S2. Continuation. III. 2-TIR copies

TAG	Scaffold	Start	End	Subfamily	TSD1	TSD2	Contig_1	Start	End	Total_length	TIR1 length	TIR2 length	TIR identity
6433-41007C	6433	41007	42966	C	ATACAAC	ATACAAC	8990<br>8989	1<br>4387	1321<br>5010	929	335	339	98.2
6473-11762C	6473	11762829	11764731	C	ATTTGAA	ATTTGAA	9647	4614	6516	1903	578	578	100
6500-31884C	6500	31884435	31886401	C	ATACTAC	ATACTAC	10790	49101	51067	1967	556	494	81.6
6540-61321C	6540	6132112	6133394	C	GTCTGGC	GTCTGGC	10985	125776	127058	1283	18	18	100
6680-24265C	6680	24265741	24266577	C	GTTCGGC	GTTCGGC	11684	12780	13616	837	133	134	94
6482-26902q	6482	269026	270625	Chimeric E/C	GTGATAT	AATACAC	9832	28557	30156	1600	577	565	67
6500-30179q	6500	30179877	30181717	Chimeric F/C	GTAGTAT	CGTAGAT	10737	2699	4539	1841	432	566	53.9
6500-30733q	6500	30733241	30734538	Chimeric D/F	no	TCTTTGG	10753	9551	10848	1298	399	535	60.5
6540-55852q	6540	558528	561650	Chimeric E/F	-	ATTTTAG	10925	93044	96166	2443	897	1050	43.5
6541-16186q	6541	1618681	1620248	Chimeric F/C	CTTTTAG	GTAACAC	11252	5173	6740	1568	754	526	80.7
6541-16912q	6541	1691275	1695391	Chimeric F/C	ATATAAC	GTCTTAA	11256	5973	10089	1865	441	454	76.5
4124-318E	4124	318	3097	E	GTAGTAA	GTAGTAA	4796<br>4795	1<br>318	3832<br>880	1866	676	559	99.1
4198-1393E	4198	1393	3341	E	GCTATAC	GCTATAC	4932<br>4931	1<br>1393	914<br>1464	1949	72	72	100
4502-5732E	4502	5732	6311	E	GTTGTAT	CCTTAAT	5475	5732	6311	580	195	195	95.9
6115-956E	6115	956	2582	E	ATATGGC	ATATGGC	7618	956	2582	1927	477	478	96.4
6482-45393E	6482	453934	454728	E	GTCAGAC	GTCAGAC	9840	16922	17716	795	53	53	98.1
6498-19996E	6498	1999631	2001782	E	ATATAAG	ATATAAG	10352	110278	112924	697	145	145	99.3
6500-31360E	6500	31360321	31362480	E	-	GTTTTAT	10770	81508	83667	2160	481	446	74.4
1776-3477F	1776	3477	4795	F	GAAGAAC	-	1899	3477	4170	1319	291	291	90.8
3792-475F	3792	475	3832	F	GTACCGC	GTACCGC	4241	475	3832	2193	846	1075	98.1
6473-16293F	6473	16293472	16295724	F	ATACAAT	ATTACAC	9762<br>9763	19387<br>1	20553<br>656	2253	651	650	99.7
6482-21925F	6482	2192546	2194452	F	ATTTGAT	ATTTGAT	9896	8068	9974	1907	812	812	100
6482-25792F	6482	2579294	2581638	F	ATTGAGT	ATTGAGT	9911	1113	3457	2345	1032	1032	99.7
6496-25846F	6496	25846816	25848819	F	ACTCTAT	ACTCTAT	10271<br>10270	1<br>145320	1156<br>146046	2004	727	727	100

Table S2. Continuation. III. 2-TIR copies

TAG	Scaffold	Start	End	Subfamily	TSD1	TSD2	Contig_1	Start	End	Total_length	TIR1 length	TIR2 length	TIR identity
6497-23418F	6497	234188	236613	F	GTACCGC	GTACCGC	10309	10744	13169	2426	1147	1153	98.8
6498-18188F	6498	1818872	1820002	F	ATCAAAT	GTGAAAC	13050	8248	9378	1032	23	23	91.3
6498-29668F	6498	2966893	2969965	F	GTAATAG	-	10404	26506	29578	2137	665	646	82.8
6498-32815F	6498	3281538	3283440	F	GTAGTAT	GTAGTAT	10415	25282	27184	1903	810	810	99.8
6500-30329F	6500	30329586	30332946	F	CTATAAC	TACATAT/TGCTAAT	10741	55570	58930	3361	985	1214	97.8
6500-30494F	6500	30494802	30496424	F	ATTTTAC	TTACGCA	10742	42243	43865	1344	396	502	91.7
6500-30596F	6500	30596827	30599409	F	GTCGTGG	GTCGTGG	10744	15274	17856	2583	1264	1038	99.3
6500-30684F	6500	30684259	30686266	F	ATAGCGT	TTGAACC	10751	6539	8546	2008	753	957	97.4
6500-30873F	6500	30873698	30875357	F	GTTATGC	GTTATGC	10758	55001	56660	1660	485	626	91.7
6500-30976F	6500	30976506	30978415	F	ATAGTAG	ATAGTAG	10762<br>10761	1<br>9087	940<br>10031	1910	944	760	93.1
6500-31107F	6500	31107017	31109152	F	CTTAAAT	CTTAAAT	10764<br>10765	53125<br>1	53803<br>1465	2136	677	679	99.8
6500-31694F	6500	31694898	31696939	F	no	GTATCAG	10387	1	2042	2042	1020	892	99.5
6540-56432F	6540	564326	566215	F	TGTACAT	ATGTACA	10925	98843	100731	1889	803	806	99
6540-79670F	6540	796704	798194	F	GTTCGTG	CCAGACA	10931<br>10930	1<br>17100	676<br>17422	1491	108	109	30.2
6540-57500F	6540	5750063	5751976	F	CTTTAAC	CTTTAAC	10984	336595	338508	1914	892	892	100
6540-33286F	6540	33286261	33288174	F	ATAAAAA	ATAAAAA	11176	117065	118978	1914	892	892	100
							11193	1	975				
6541-17831F	6541	178316	180001	F	ATAAGAC	ATAAGAC	11192	15669	16353	1686	682	683	98.7
6541-10035F	6541	1003587	1007838	F	ATATAAG	CCCATAT	11229	9895	14146	1876	712	688	93
6541-12491F	6541	1249195	1251094	F	CTAATAT	CTAATAT	11238	17034	18933	1900	811	811	99.1
6541-15113F	6541	1511326	1513666	F	CTTTGTG	CTTTGTG	11249<br>11248	1<br>12735	1329<br>13721	2341	751	964	95.3
6680-24420F	6680	24420206	24421090	F	GTAGTAT	GTAGTAT	11687	39553	40437	885	86	86	93

Table S3. Continuation. III. 2-TIR copies

TAG	Scaffold	Start	End	Subfamily	TSD1	TSD2	Contig_1	Start	End	Total_length	TIR1 length	TIR2 length	TIR identity
6680-24422F	6680	24422223	24425258	F	GTACACA	GTACACA	11687	41570	44605	1451	335	335	97
6498-95069X	6498	950693	951185	X	TCCATAT	TCCATAT	10339	90483	90975	493	90	88	98.8
6498-29033X	6498	2903343	2904985	X	CTTATAT	CTTATAT	10400	3858	5500	1643	423	423	100
6500-29395X	6500	29395284	29396934	X	ATAATAC	TATAAAC	10722	198610	200260	1651	423	423	99.1

Table S2. Continuation. IV. 2 Recombinant TIR or 2 longer TIR copies.

TAG	Scaffold	Start	End	Subfamily	TSD1	TSD2	Contig_1	Start	End	Total_length	TIR1 length	TIR2 length	TIR identity	TPase
6498-21770C	6498	2177013	2181005	C	GTTGAGC	GTTGAGC	10367	3528	7520	3119	1107	1107	99.1	599
6500-31083C	6500	31083091	31089863	C	TTTATAT	TTTATAT	10764	29199	35971	2469	815	715	86.4	57
6500-31371C	6500	31371001	31374119	C	ATAGTAG	CTACTAT	10770	92188	95306	3119	1107	1107	98.7	602
6500-29973q	6500	29973001	29975284	Chimeric F/X	TAGGTAA	ATACAAC	10735	14871	17154	2284	590	746	49.8	0
6500-30183q	6500	30183437	30184591	Chimeric F/X	ATAATAC	CATATAT	10737	6259	7413	1155	449	714	59.3	0
6541-99710q	6541	997100	998743	Chimeric E/F	ACCATAC	GTACAGC	11229	3408	5051	1644	46	47	87.2	0
6680-24427q	6680	24427759	24434511	Chimeric E/X	TTTGGGT	ATGTTAA	11687<br>11688	47106<br>1	52345<br>552	2643	555	552	85.5	0
6680-24440q	6680	24440484	24442275	Chimeric E/X	TTTTGGT	ATGTTAA	11688	6525	8316	1792	608	606	86.8	0
6540-10358D	6540	1035815	1037361	D	ATTGGGG	ATTGGGG	10940	39859	41405	1547	458	458	96.5	0
6680-24069D	6680	24069812	24072777	D	TTATGAG	TTATGAG	11679	95650	98615	2174	1012	1014	98.9	0
4503-1178E	4503	1178	7564	E	TCGTGAC	TCGTGAC	5476	1178	7564	1991	954	979	98.1	0
6395-2229E	6395	2229	6819	E	CTATAAC	CTATAAC	8783	2229	6819	2151	991	1028	95.6	0
6473-10080E	6473	1008070	1010341	E	GCGCTGA	GCGCTGA	9253<br>9252	1<br>9335	1207<br>10394	2272	1002	1002	99.7	0
6482-36252E	6482	362528	364455	E	-	GTTTAC	9834<br>9835	20890<br>327	25520<br>959	1928	741	741	98.4	0
6496-15292E	6496	15292514	15294879	E	TAAGTGG	TAAGTGG	10177	520481	522846	2366	1075	1075	100	0
6498-95355E	6498	953555	955919	E	GCCAAAG	GCCAAAG	10339	93345	95709	2365	1075	1074	99.9	0
6498-29938E	6498	2993866	2995242	E	CTTGTAC	CTTGTAC	10405	19460	20836	1377	193	193	100	0
6500-29804E	6500	29804958	29807163	E	TCATTAC	TCATTAC	10727	44829	47034	2206	1029	1027	99.6	0
6500-30306E	6500	30306361	30308225	E	GTGGTAT	GTGGTAT	10741	32345	34209	1865	645	678	96	0
6500-30702E	6500	30702790	30704984	E	TGTATAC	TGTATAC	10751	25070	27264	2195	1021	1026	99.3	0
6500-31202E	6500	31202553	31204509	E	ACATCAA	ACATCAA	10766<br>10767	25837<br>1	26845<br>913	1957	832	819	96.6	0
6500-31506E	6500	31506397	31509717	E	GTAAAAA	GTAAAAC	10774	18407	21727	3321	1048	1043	97.4	0
6500-31516E	6500	31516211	31518161	E	ATACTAG	GTACAGG	10774<br>10775	28221<br>1	29239<br>907	1951	962	906	92.2	0
6500-31920E	6500	31920296	31922494	E	TCGAAAC	TCGAAAC	10790	84962	87962	2199	1028	1022	98.2	0
6500-32268E	6500	32268286	32271024	E	ATTATAG	ATTATAG	10803	46034	48772	2184	1016	1017	99.2	0

Table S2. Continuation. IV. Recombinant TIR or 2 longer TIR copies (continuation).

TAG	Scaffold	Start	End	Subfamily	TSD1	TSD2	Contig_1	Start	End	Total_length	TIR1 length	TIR2 length	TIR identity	Tpase
6540-11650E	6540	1165033	1168349	E	GATACAC	GATACAC	10945	2811	6127	2180	998	1034	96.2	0
6540-13720E	6540	1372066	1373808	E	ATATAAT	ATATAAT	10949	9737	1479	1743	771	771	99.2	0
6540-14510E	6540	14510521	14512886	E	CTTTTGT	CTTTTGT	11044	136297	138662	2366	1075	1075	100	0
6540-31163E	6540	31163990	31166355	E	CTTAAAC	TTAGTGC	11157	380472	382837	2366	1075	1075	99.3	0
6541-10420E	6541	1042036	1043771	E	TTAATGC	TTAATGC	11229	48344	50079	1736	769	971	96.5	0
6541-10885E	6541	1088506	1090501	E	ATAGAGC	ATAGAGC	11232<br>11231	1<br>24008	1014<br>24917	1996	910	915	98.1	0
6541-20142E	6541	2014221	2016575	E	GTATCAA	GTATCAA	11267	12789	15143	1794	613	623	98.1	0
6328-16507F	6328	1650720	1653101	F	GTGCAGC	GTGCAGC	8189	12012	14393	2382	894	894	99.9	0
6496-23195F	6496	23195067	23197892	F	GTATTTT	GTATTTT	10246	221912	224737	2826	1116	1116	99.6	211
6500-29864F	6500	29864896	29868109	F	GTATTAT	GTATTAT	10727	104767	107980	3214	1260	1241	95.2	0
6500-30351X	6500	30351497	30353286	X	CTATAAC	CTATAAC	10741	77481	79270	1790	606	606	99.5	0
6680-24283X	6680	24283772	24285562	X	GCTAAAG	ATTAAAG	11684	30811	32601	1791	606	606	98.3	0
6680-24520X	6680	24520907	24522561	X	ATAAGAC	ATAAGAC	11693	28363	30017	1655	548	566	95.2	0
6680-24538X	6680	24538620	24540276	X	GTTACGG	GTTACGG	11694	11280	12936	1657	550	566	95.1	0

Table S2. Continuation. V. Solo-TIR copies

TAG	Scaffold	Start	End	Subfamily	TSD1	Contig_1	Start	End	Total_length
4159-2383C	4159	2383	3089	C	no	4862	93	745	707
4315-36359C	4315	36359	37136	C	ATTTAGG	5157	597	1374	778
6475-6418C	6475	6418	6949	C	GTTATGC	9793	6418	6949	532
6500-29798C	6500	29798764	29799506	C	-	10727	39377	38635	743
6540-59683C	6540	596833	597648	C	GTTGAAC	10925	131349	132164	816
6540-13434C	6540	13434776	13435834	C	ATACCC	11040	106510	108568	1059
3967-5428D	3967	5428	5995	D	GTATTGA	4504	449	1016	568
4302-1710D	4302	1710	2167	D	TTCACGA	5129	23	480	458
5820-1010D	5820	1010	1528	D	GCTTTAT	7167	1010	1528	519
6115-1D	6115	1	291	D	ATTTAAG	7618	1	291	291
6422-3900D	6422	3900	4471	D	TTGATGT	8929	3900	4471	572
6439-76259D	6439	76259	76829	D	GATAAAT	9016	2195	2765	571
6482-25268D	6482	2526809	2527340	D	CTACTAC	9907	14465	14996	532
6498-25609D	6498	2560957	2561558	D	TCATAAC	10383	46934	47262	602
6500-30590D	6500	30590766	30591289	D	CTTCTAG	10744	9213	9736	524
6541-24219D	6541	2421943	2422508	D	ATCGTTC ?	11283	10937	11502	885
3878-2398E	3878	2398	2973	E	ATAATAG	4340	2398	2973	576
4315-35763E	4315	35763	36259	E	GCGCAAC	9252	9355	9851	497
4552-6419E	4552	6419	7232	E	CCATAAA	5552	3434	4074	814
4621-5761E	4621	5761	6344	E	CTTCTAG	5655	474	1057	584
6070-5751E	6070	5751	6783	E	TCGTGAC	7517	5751	6783	1033
6320-38399E	6320	38399	38982	E	GTTCTGC	8092	3568	4151	584
6329-49349E	6329	49349	50504	E	TTACTAC	8308	1	1156	1156
6404-43168E	6404	43168	43745	E	GTTGAAG	8826	832	1409	578
6498-24079E	6498	2407995	2408421	E	GTTCTAT	10376	26216	26642	427
6498-26098E	6498	2609857	2611989	E	GTTTTGA	10385	7751	8354	1149
6498-28362E	6498	2836237	2836700	E	TTGAAAG	10397	7407	7870	464

Table S2. Continuation. V. Solo-TIR copies (continuation)

TAG	Scaffold	Start	End	Subfamily	TSD1	Contig_1	Start	End	Total_length
6498-31200E	6498	3120041	3121220	E	CAGTTGG	10408	4700	5879	1180
6500-31339E	6500	31339017	31339980	E	ATATTAT	10770	60532	61167	964
6500-31499E	6500	31499776	31500354	E	CATTAAC	10774	11786	12364	579
6500-31817E	6500	31817847	31818422	E	GTCACGA	10789	10934	11509	576
6540-75029E	6540	750291	750804	E	ACCATAC	10928	42449	42962	514
6540-89813E	6540	898138	898666	E	CTTATAT	10934	44264	44792	529
6540-10067E	6540	1006735	1007752	E	no	10940	10779	11429	1018
6680-23161E	6680	23161869	23162539	E	ATATAAG	11659	26002	26672	821
6498-17302F	6498	1730250	1731243	F	CTGTTAC	10349	8459	9452	994
6498-23818F	6498	2381827	2382890	F	ATTAAAT	10376	48	1111	1064
6498-25144F	6498	2514476	2515268	F	GCAAAAT	10383	180	972	793
6498-25221F	6498	2522128	2522920	F	GCAAAAT	10383	7832	8624	793
6498-27869F	6498	2786970	2787863	F	ATCATAT	10394	5133	6204	894
6498-30224F	6498	3022490	3023060	F	no	10406	18750	19320	571
6500-29965F	6500	29965273	29966306	F	GTAGTAC	10735	7404	7734	1034
6500-29967F	6500	29967217	29968358	F	GTGCTAT	10735	9087	10228	1142
6500-29976F	6500	29976999	29977829	F	TAAGTAC	10735	18869	19699	831
6500-30981F	6500	30981230	30981940	F	no	10762	3155	4465	711
6500-31888F	6500	31888888	31889062	F	GTATAAT	10790	53554	53728	175
6500-32144F	6500	32144419	32145123	F	TTATAAT	10797	25558	26210	705
6540-32266F	6540	322669	323530	F	TCACTAC	10921	4012	4873	862
6540-46643F	6540	466436	467246	F	TTTAAAG	10925	952	1762	811
6540-62798F	6540	627982	628716	F	ATATTGA	10925	162498	163232	735
6540-69428F	6540	694288	695126	F	GTTCAGA	10927	19474	20312	839
6540-75429F	6540	754292	755195	F	GTAGTAT	10928	46450	47353	904
6540-10727F	6540	1072704	1073438	F	CTTATAT	10941	6593	7327	735
6540-73206F	6540	7320643	7321437	F	GTGGAAC	10998	72162	72956	795
6541-83575F	6541	835755	836619	F	ATTATAT	11224	13291	14155	865
6541-10932F	6541	1093209	1093801	F	GTACAGA	11232	3722	4314	593

Table 2. Continuation. V. Solo-TIR copies (continuation)

TAG	Scaffold	Start	End	Subfamily	TSD1	Contig_1	Start	End	Total_length
6541-24225F	6541	2422509	2423290	F	GTTCAGG	11283	11503	12284	782
6680-23160F	6680	23160719	23161539	F	GTTATAA	11659	24852	25672	671
6680-23219F	6680	23219687	23220569	F	CTCTAAC	11661	17802	18684	883
6680-23825F	6680	23825194	23825885	F	GCAGAAA	11672	39965	40656	692
6680-24145F	6680	24145587	24146659	F	GTACAGA	11680	28664	29736	1073
6493-38387X	6493	38387	39006	X	GTAATAT	9982	1	620	620
6500-31891X	6500	31891331	31891606	X	no	10790	55997	56272	276
6540-72269X	6540	722695	723349	X	ATATGAA	10928	14931	15507	655

Table S**3**. Chromosomal distribution of *Galileo* copies in *D. mojavensis*.

CAF1_scaffold	Galileo_start	Galileo_end	Galileo_Group	Galileo_Type	Galileo_length	GenBank Scaffold Acc	Scaffold_length_(bp)	Chr_arm
6328	1650720	1653101	F	2TIR	2382	CH933812.1	4453435	X
6473	1008070	1010341	E	Longer_2TIR	2272	CH933810.1	16943266	X
6473	11762829	11764731	C	2TIR	1903	CH933810.1	16943266	X
6473	16293472	16295724	F	2TIR	2253	CH933810.1	16943266	X
6482	269026	270625	Chimeric	2TIR	1600	CH933815.1	2735782	X
6482	362528	364455	E	Longer_2TIR	1928	CH933815.1	2735782	X
6482	453934	454728	E	2TIR	795	CH933815.1	2735782	X
6482	608936	612509	F	NC_DD	3574	CH933815.1	2735782	X
6482	614003	617184	D	NC_DD	3182	CH933815.1	2735782	X
6482	617185	621442	D	NC_DD	3390	CH933815.1	2735782	X
6482	2192546	2194452	F	2TIR	1907	CH933815.1	2735782	X
6482	2526809	2527340	D	SOLO	532	CH933815.1	2735782	X
6482	2579294	2581638	F	2TIR	2345	CH933815.1	2735782	X
6496	15292514	15294879	E	Longer_2TIR	2366	CH933808.1	26866924	5
6496	23195067	23197892	F	NC_DD	2826	CH933808.1	26866924	5
6496	25846816	25848819	F	2TIR	2004	CH933808.1	26866924	5
6498	950693	951185	X	2TIR	493	CH933813.1	3408170	6
6498	953555	955919	E	Longer_2TIR	2365	CH933813.1	3408170	6
6498	1730250	1731243	F	SOLO	994	CH933813.1	3408170	6
6498	1818872	1820002	F	2TIR	1032	CH933813.1	3408170	6
6498	1999631	2001782	E	2TIR	697	CH933813.1	3408170	6
6498	2177013	2181005	C	Longer_2TIR	3119	CH933813.1	3408170	6
6498	2253149	2269701	F	NC	4036	CH933813.1	3408170	6
6498	2381827	2382890	F	SOLO	1064	CH933813.1	3408170	6
6498	2386095	2392524	D	NC	5721	CH933813.1	3408170	6
6498	2407995	2408421	E	SOLO	427	CH933813.1	3408170	6
6498	2514476	2515268	F	SOLO	793	CH933813.1	3408170	6
6498	2522128	2522920	F	SOLO	793	CH933813.1	3408170	6

Table S3. Chromosomal distribution of *Galileo* copies in *D. mojavensis* (continuation).

CAF1_scaffold	Galileo_start	Galileo_end	Galileo_Group	Galileo_Type	Galileo_length	GenBank Scaffold Acc	Scaffold_length_(bp)	Chr_arm
6498	2541172	2544793	X	NC_DD	2423	CH933813.1	3408170	6
6498	2560957	2561558	D	SOLO	602	CH933813.1	3408170	6
6498	2609857	2611989	E	SOLO	1149	CH933813.1	3408170	6
6498	2786970	2787863	F	SOLO	894	CH933813.1	3408170	6
6498	2836237	2836700	E	SOLO	464	CH933813.1	3408170	6
6498	2903343	2904985	X	2TIR	1643	CH933813.1	3408170	6
6498	2966893	2969965	F	2TIR	2137	CH933813.1	3408170	6
6498	2993866	2995242	E	2TIR	1377	CH933813.1	3408170	6
6498	3022490	3023060	F	SOLO	571	CH933813.1	3408170	6
6498	3120041	3121220	E	SOLO	1180	CH933813.1	3408170	6
6498	3281538	3283440	F	2TIR	1903	CH933813.1	3408170	6
6500	29395284	29396934	X	2TIR	1651	CH933807.1	32352404	3
6500	29798764	29799506	C	SOLO	743	CH933807.1	32352404	3
6500	29804958	29807163	E	Longer_2TIR	2206	CH933807.1	32352404	3
6500	29864896	29868109	F	Longer_2TIR	3214	CH933807.1	32352404	3
6500	29965273	29966306	F	SOLO	1034	CH933807.1	32352404	3
6500	29967217	29968358	F	SOLO	1142	CH933807.1	32352404	3
6500	29973001	29975284	Chimeric	2TIR	2284	CH933807.1	32352404	3
6500	29976999	29977829	F	SOLO	831	CH933807.1	32352404	3
6500	30179877	30181717	Chimeric	2TIR	1841	CH933807.1	32352404	3
6500	30183437	30184591	Chimeric	2TIR	1155	CH933807.1	32352404	3
6500	30306361	30308225	E	Longer_2TIR	1865	CH933807.1	32352404	3
6500	30329586	30332946	F	2TIR	3361	CH933807.1	32352404	3
6500	30351497	30353286	X	Longer_2TIR	1790	CH933807.1	32352404	3
6500	30494802	30496424	F	2TIR	1344	CH933807.1	32352404	3
6500	30590766	30591289	D	SOLO	524	CH933807.1	32352404	3

Table S3. Chromosomal distribution of *Galileo* copies in *D. mojavensis* (continuation).

CAF1_scaffold	Galileo_start	Galileo_end	Galileo_Group	Galileo_Type	Galileo_length	GenBank Scaffold Acc	Scaffold_length_(bp)	Chr_arm
6500	30596827	30599409	F	2TIR	2583	CH933807.1	32352404	3
6500	30684259	30686266	F	2TIR	2008	CH933807.1	32352404	3
6500	30702790	30704984	E	Longer_2TIR	2195	CH933807.1	32352404	3
6500	30733241	30734538	Chimeric	2TIR	1298	CH933807.1	32352404	3
6500	30856283	30862823	C	NC	5989	CH933807.1	32352404	3
6500	30873698	30875357	F	2TIR	1660	CH933807.1	32352404	3
6500	30976506	30978415	F	2TIR	1910	CH933807.1	32352404	3
6500	30981230	30981940	F	SOLO	711	CH933807.1	32352404	3
6500	31083091	31089863	C	NC_DD	2469	CH933807.1	32352404	3
6500	31107017	31109152	F	2TIR	2136	CH933807.1	32352404	3
6500	31202553	31204509	E	Longer_2TIR	1957	CH933807.1	32352404	3
6500	31288762	31295303	C	NC	5836	CH933807.1	32352404	3
6500	31339017	31339980	E	SOLO	964	CH933807.1	32352404	3
6500	31360321	31362480	E	2TIR	2160	CH933807.1	32352404	3
6500	31371001	31374119	C	Longer_2TIR	3119	CH933807.1	32352404	3
6500	31458921	31465167	D	NC	4130	CH933807.1	32352404	3
6500	31499776	31500354	E	SOLO	579	CH933807.1	32352404	3
6500	31506397	31509717	E	Longer_2TIR	3321	CH933807.1	32352404	3
6500	31516211	31518161	E	Longer_2TIR	1951	CH933807.1	32352404	3
6500	31694898	31696939	F	2TIR	2042	CH933807.1	32352404	3
6500	31817847	31818422	E	SOLO	576	CH933807.1	32352404	3
6500	31884435	31886401	C	2TIR	1967	CH933807.1	32352404	3
6500	31888888	31889062	F	SOLO	175	CH933807.1	32352404	3
6500	31891331	31891606	X	SOLO	276	CH933807.1	32352404	3
6500	31920296	31922494	E	Longer_2TIR	2199	CH933807.1	32352404	3
6500	31981325	31986443	C	NC_DD	5119	CH933807.1	32352404	3
6500	32144419	32145123	F	SOLO	705	CH933807.1	32352404	3
6500	32268286	32271024	E	Longer_2TIR	2184	CH933807.1	32352404	3

Table S3. Chromosomal distribution of *Galileo* copies in *D. mojavensis* (continuation).

CAF1_scaffold	Galileo_start	Galileo_end	Galileo_Group	Galileo_Type	Galileo_length	GenBank Scaffold Acc	Scaffold_length_(bp)	Chr_arm
6540	322669	323530	F	SOLO	862	CH933806.1	34148556	2
6540	414493	419539	X	NC	5047	CH933806.1	34148556	2
6540	466436	467246	F	SOLO	811	CH933806.1	34148556	2
6540	558528	561650	Chimeric	2TIR	2443	CH933806.1	34148556	2
6540	564326	566215	F	2TIR	1889	CH933806.1	34148556	2
6540	596833	597648	F	SOLO	816	CH933806.1	34148556	2
6540	627982	628716	F	SOLO	735	CH933806.1	34148556	2
6540	694288	695126	F	SOLO	839	CH933806.1	34148556	2
6540	722695	723349	X	SOLO	655	CH933806.1	34148556	2
6540	750291	750804	E	SOLO	514	CH933806.1	34148556	2
6540	754292	755195	F	SOLO	904	CH933806.1	34148556	2
6540	796704	798194	F	2TIR	1491	CH933806.1	34148556	2
6540	898138	899882	Chimeric	SOLO	1285	CH933806.1	34148556	2
6540	1006735	1007752	E	SOLO	1018	CH933806.1	34148556	2
6540	1035815	1037361	D	2TIR	1547	CH933806.1	34148556	2
6540	1072704	1073438	F	SOLO	735	CH933806.1	34148556	2
6540	1165033	1168349	E	Longer_2TIR	2180	CH933806.1	34148556	2
6540	1175880	1182997	D	NC	5433	CH933806.1	34148556	2
6540	1372066	1373808	E	Longer_2TIR	1743	CH933806.1	34148556	2
6540	5750063	5751976	F	2TIR	1914	CH933806.1	34148556	2
6540	6132112	6133394	C	2TIR	1283	CH933806.1	34148556	2
6540	7320643	7321437	F	SOLO	795	CH933806.1	34148556	2
6540	13434776	13435834	C	SOLO	1059	CH933806.1	34148556	2
6540	14510521	14512886	E	Longer_2TIR	2366	CH933806.1	34148556	2
6540	31163990	31166355	E	Longer_2TIR	2366	CH933806.1	34148556	2
6540	33286261	33288174	F	2TIR	1914	CH933806.1	34148556	2
6541	178316	180001	F	2TIR	1686	CH933817.1	2543558	X

Table S3. Chromosomal distribution of *Galileo* copies in *D. mojavensis* (continuation).

CAF1_scaffold	Galileo_start	Galileo_end	Galileo_Group	Galileo_Type	Galileo_length	GenBank Scaffold Acc	Scaffold_length_(bp)	Chr_arm
6541	835755	836619	F	SOLO	865	CH933817.1	2543558	X
6541	997100	998743	Chimeric	2TIR	1644	CH933817.1	2543558	X
6541	1003587	1007838	F	2TIR	1876	CH933817.1	2543558	X
6541	1042036	1043771	E	Longer_2TIR	1736	CH933817.1	2543558	X
6541	1088506	1090501	E	Longer_2TIR	1996	CH933817.1	2543558	X
6541	1093209	1093801	F	SOLO	593	CH933817.1	2543558	X
6541	1141978	1149130	Chimeric	NC	6239	CH933817.1	2543558	X
6541	1249195	1251094	F	2TIR	1900	CH933817.1	2543558	X
6541	1511326	1513666	F	2TIR	2341	CH933817.1	2543558	X
6541	1618681	1620248	Chimeric	2TIR	1568	CH933817.1	2543558	X
6541	1644296	1649755	D	NC	5460	CH933817.1	2543558	X
6541	1691275	1695391	Chimeric	2TIR	1865	CH933817.1	2543558	X
6541	2014221	2016575	E	2TIR	1794	CH933817.1	2543558	X
6541	2421943	2422508	D	SOLO	885	CH933817.1	2543558	X
6541	2422509	2423290	F	SOLO	782	CH933817.1	2543558	X
6680	23160719	23161539	F	SOLO	671	CH933809.1	24764193	4
6680	23161869	23162539	E	SOLO	821	CH933809.1	24764193	4
46680	23219687	23220569	F	SOLO	883	CH933809.1	24764193	4
6680	23825194	23825885	F	SOLO	692	CH933809.1	24764193	4
6680	24069812	24072777	D	Longer_2TIR	2174	CH933809.1	24764193	4
6680	24145587	24146659	F	SOLO	1073	CH933809.1	24764193	4
6680	24265741	24266577	C	2TIR	837	CH933809.1	24764193	4
6680	24283772	24285562	X	Longer_2TIR	1791	CH933809.1	24764193	4
6680	24420206	24421090	F	2TIR	885	CH933809.1	24764193	4
6680	24422223	24425258	F	2TIR	1451	CH933809.1	24764193	4
6680	24427759	24434511	Chimeric	2TIR	2643	CH933809.1	24764193	4
6680	24440484	24442275	Chimeric	2TIR	1792	CH933809.1	24764193	4
6680	24520907	24522561	X	Longer_2TIR	1655	CH933809.1	24764193	4
6680	24538620	24540276	X	Longer_2TIR	1657	CH933809.1	24764193	4

Table S**4**. Intrachromosomal distribution of *Galileo* elements.
Chromosome X

Scaffold	Scaffold_length	GenBank_acc	Chr_arm	Galileo_start	Galileo_end	Galileo_subfam	Galileo_type	Galileo_length	Chr_region
6482	2735782	CH933815.1	X	269026	270625	Chimeric	2TIR	1600	Central
6482	2735782	CH933815.1	X	362528	364455	E	Longer_2TIR	1928	Central
6482	2735782	CH933815.1	X	453934	454728	E	2TIR	795	Central
6482	2735782	CH933815.1	X	608936	612509	F	NC_DD	3574	Central
6482	2735782	CH933815.1	X	614003	617184	D	NC_DD	3182	Central
6482	2735782	CH933815.1	X	617185	621442	D	NC_DD	3390	Central
6482	2735782	CH933815.1	X	2192546	2194452	F	2TIR	1907	Central
6482	2735782	CH933815.1	X	2526809	2527340	D	SOLO	532	2
6482	2735782	CH933815.1	X	2579294	2581638	F	2TIR	2345	2

Scf_6482	Proportion	Region Start	Region End	*Galileo* Obs	*Galileo* Exp		
Region 1	10.00%	1	273578	0	0.9	Chi square test	
Central region	80.00%	273579	2462203	7	7.2	**P-val=**	**0.3246524674**
Region 2	10.00%	2462204	2735782	2	0.9		
				9	9		

Table S4. Intrachromosomal distribution of *Galileo* elements (continuation).

Scaffold	GenBank_acc	Chr_arm	Scaffold_length	Galileo_start	Galileo_end	Galileo_subfam	Galileo_type	Galileo_length	Region
6541	CH933817.1	X	2543558	178316	180001	F	2TIR	1686	1
6541	CH933817.1	X	2543558	835755	836619	F	SOLO	865	Central
6541	CH933817.1	X	2543558	997100	998743	Chimeric	2TIR	1644	Central
6541	CH933817.1	X	2543558	1003587	1007838	F	2TIR	1876	Central
6541	CH933817.1	X	2543558	1042036	1043771	E	Longer_2TIR	1736	Central
6541	CH933817.1	X	2543558	1088506	1090501	E	Longer_2TIR	1996	Central
6541	CH933817.1	X	2543558	1093209	1093801	F	SOLO	593	Central
6541	CH933817.1	X	2543558	1141978	1149130	Chimeric	NC	6239	Central
6541	CH933817.1	X	2543558	1249195	1251094	F	2TIR	1900	Central
6541	CH933817.1	X	2543558	1511326	1513666	F	2TIR	2341	Central
6541	CH933817.1	X	2543558	1618681	1620248	Chimeric	2TIR	1568	Central
6541	CH933817.1	X	2543558	1644296	1649755	D	NC	5460	Central
6541	CH933817.1	X	2543558	1691275	1695391	Chimeric	2TIR	1865	Central
6541	CH933817.1	X	2543558	2014221	2016575	E	2TIR	1794	Central
6541	CH933817.1	X	2543558	2421943	2422508	D	SOLO	885	2
6541	CH933817.1	X	2543558	2422509	2423290	F	SOLO	782	2

Scf_6541	Proportion	Region Start	Region End	*Galileo* Obs	*Galileo* Exp		
Region 1	10.00%	1	254356	1	1.6	Chi square test	
Central region	80.00%	254357	2289202	13	12.8	**P-val=**	**0.8486889772**
Region 2	10.00%	2289203	2543558	2	1.6		
				16	16		

Table S4. Intrachromosomal distribution of *Galileo* elements (continuation).
Chromosome 2

Scaffold	GenBank_acc	Chr_arm	Scaffold_length	Galileo_start	Galileo_end	Galileo_subfam	Galileo_type	Galileo_length	Region
6540	CH933806.1	2	34148556	322669	323530	F	SOLO	862	1
6540	CH933806.1	2	34148556	414493	419539	X	NC	5047	1
6540	CH933806.1	2	34148556	466436	467246	F	SOLO	811	1
6540	CH933806.1	2	34148556	558528	561650	Chimeric	2TIR	2443	1
6540	CH933806.1	2	34148556	564326	566215	F	2TIR	1889	1
6540	CH933806.1	2	34148556	596833	597648	F	SOLO	816	1
6540	CH933806.1	2	34148556	627982	628716	F	SOLO	735	1
6540	CH933806.1	2	34148556	694288	695126	F	SOLO	839	1
6540	CH933806.1	2	34148556	722695	723349	X	SOLO	655	1
6540	CH933806.1	2	34148556	750291	750804	E	SOLO	514	1
6540	CH933806.1	2	34148556	754292	755195	F	SOLO	904	1
6540	CH933806.1	2	34148556	796704	798194	F	2TIR	1491	1
6540	CH933806.1	2	34148556	898138	899882	Chimeric	SOLO	1285	1
6540	CH933806.1	2	34148556	1006735	1007752	E	SOLO	1018	1
6540	CH933806.1	2	34148556	1035815	1037361	D	2TIR	1547	1
6540	CH933806.1	2	34148556	1072704	1073438	F	SOLO	735	1
6540	CH933806.1	2	34148556	1165033	1168349	E	Longer_2TIR	2180	1
6540	CH933806.1	2	34148556	1175880	1182997	D	NC	5433	1
6540	CH933806.1	2	34148556	1372066	1373808	E	Longer_2TIR	1743	1
6540	CH933806.1	2	34148556	5750063	5751976	F	2TIR	1914	Central
6540	CH933806.1	2	34148556	6132112	6133394	C	2TIR	1283	Central
6540	CH933806.1	2	34148556	7320643	7321437	F	SOLO	795	Central
6540	CH933806.1	2	34148556	13434776	13435834	C	SOLO	1059	Central
6540	CH933806.1	2	34148556	14510521	14512886	E	Longer_2TIR	2366	Central
6540	CH933806.1	2	34148556	31163990	31166355	E	Longer_2TIR	2366	2
6540	CH933806.1	2	34148556	33286261	33288174	F	2TIR	1914	2

Table S4. Intrachromosomal distribution of *Galileo* elements (continuation).

Scf_6540	Proportion	Region Start	Region End	Galileos Obs	Galileos Exp		
Centromeric region 1	10.00%	1	3414856	19	2.6	Chi square test	**7.956133869452 44E-026**
Central region	80.00%	3414857	30733700	5	20.8		
Telomeric fraction 2	10.00%	30733701	34148556	2	2.6		
				26	26		

Chromosome 3

Scaffold	GenBank_acc	Chr_arm	Scaffold_length	Galileo_start	Galileo_end	Galileo_subfam	Galileo_type	Galileo_length	Region
6500	CH933807.1	3	32352404	29395284	29396934	X	2TIR	1651	1
6500	CH933807.1	3	32352404	29798764	29799506	C	SOLO	743	1
6500	CH933807.1	3	32352404	29804958	29807163	E	Longer_2TIR	2206	1
6500	CH933807.1	3	32352404	29864896	29868109	F	Longer_2TIR	3214	1
6500	CH933807.1	3	32352404	29965273	29966306	F	SOLO	1034	1
6500	CH933807.1	3	32352404	29967217	29968358	F	SOLO	1142	1
6500	CH933807.1	3	32352404	29973001	29975284	Chimeric	2TIR	2284	1
6500	CH933807.1	3	32352404	29976999	29977829	F	SOLO	831	1
6500	CH933807.1	3	32352404	30179877	30181717	Chimeric	2TIR	1841	1
6500	CH933807.1	3	32352404	30183437	30184591	Chimeric	2TIR	1155	1
6500	CH933807.1	3	32352404	30306361	30308225	E	Longer_2TIR	1865	1
6500	CH933807.1	3	32352404	30329586	30332946	F	2TIR	3361	1
6500	CH933807.1	3	32352404	30351497	30353286	X	Longer_2TIR	1790	1
6500	CH933807.1	3	32352404	30494802	30496424	F	2TIR	1344	1
6500	CH933807.1	3	32352404	30590766	30591289	D	SOLO	524	1
6500	CH933807.1	3	32352404	30596827	30599409	F	2TIR	2583	1
6500	CH933807.1	3	32352404	30684259	30686266	F	2TIR	2008	1
6500	CH933807.1	3	32352404	30702790	30704984	E	Longer_2TIR	2195	1
6500	CH933807.1	3	32352404	30733241	30734538	Chimeric	2TIR	1298	1

Table S4. Intrachromosomal distribution of *Galileo* elements (continuation).

Scaffold	GenBank_acc	Chr_arm	Scaffold_length	Galileo_start	Galileo_end	Galileo_subfam	Galileo_type	Galileo_length	Region
6500	CH933807.1	3	32352404	30856283	30862823	C	NC	5989	1
6500	CH933807.1	3	32352404	30873698	30875357	F	2TIR	1660	1
6500	CH933807.1	3	32352404	30976506	30978415	F	2TIR	1910	1
6500	CH933807.1	3	32352404	30981230	30981940	F	SOLO	711	1
6500	CH933807.1	3	32352404	31083091	31089863	C	NC_DD	2469	1
6500	CH933807.1	3	32352404	31107017	31109152	F	2TIR	2136	1
6500	CH933807.1	3	32352404	31202553	31204509	E	Longer_2TIR	1957	1
6500	CH933807.1	3	32352404	31288762	31295303	C	NC	5836	1
6500	CH933807.1	3	32352404	31339017	31339980	E	SOLO	964	1
6500	CH933807.1	3	32352404	31360321	31362480	E	2TIR	2160	1
6500	CH933807.1	3	32352404	31371001	31374119	C	Longer_2TIR	3119	1
6500	CH933807.1	3	32352404	31458921	31465167	D	NC	4130	1
6500	CH933807.1	3	32352404	31499776	31500354	E	SOLO	579	1
6500	CH933807.1	3	32352404	31506397	31509717	E	Longer_2TIR	3321	1
6500	CH933807.1	3	32352404	31516211	31518161	E	Longer_2TIR	1951	1
6500	CH933807.1	3	32352404	31694898	31696939	F	2TIR	2042	1
6500	CH933807.1	3	32352404	31817847	31818422	E	SOLO	576	1
6500	CH933807.1	3	32352404	31884435	31886401	C	2TIR	1967	1
6500	CH933807.1	3	32352404	31888888	31889062	F	SOLO	175	1
6500	CH933807.1	3	32352404	31891331	31891606	X	SOLO	276	1
6500	CH933807.1	3	32352404	31920296	31922494	E	Longer_2TIR	2199	1
6500	CH933807.1	3	32352404	31981325	31986443	C	NC_DD	5119	1
6500	CH933807.1	3	32352404	32144419	32145123	F	SOLO	705	1
6500	CH933807.1	3	32352404	32268286	32271024	E	Longer_2TIR	2184	1

Scf_6500	Proportion	Region Start	Region End	Observed	Expected		
Centromeric (1)	10.00%	29117164	32352404	43	4.3	Chi square test	
Central	80.00%	3235241	29117163	0	34.4	**P-val=**	**9.20487195758081E-085**
Telomeric (2)	10.00%	1	3235240	0	4.3		
				43	43		

Table S4. Intrachromosomal distribution of *Galileo* elements (continuation).
Chromosome 4

Scaffold	GenBank_acc	Chr_arm	Scaffold_length	Galileo_start	Galileo_end	Galileo_subfam	Galileo_type	Galileo_length	Region
6680	CH933809.1	4	24764193	23160719	23161539	F	SOLO	671	1
6680	CH933809.1	4	24764193	23161869	23162539	E	SOLO	821	1
6680	CH933809.1	4	24764193	23219687	23220569	F	SOLO	883	1
6680	CH933809.1	4	24764193	23825194	23825885	F	SOLO	692	1
6680	CH933809.1	4	24764193	24069812	24072777	D	Longer_2TIR	2174	1
6680	CH933809.1	4	24764193	24145587	24146659	F	SOLO	1073	1
6680	CH933809.1	4	24764193	24265741	24266577	C	2TIR	837	1
6680	CH933809.1	4	24764193	24283772	24285562	X	Longer_2TIR	1791	1
6680	CH933809.1	4	24764193	24420206	24421090	F	2TIR	885	1
6680	CH933809.1	4	24764193	24422223	24425258	F	2TIR	1451	1
6680	CH933809.1	4	24764193	24427759	24434511	Chimeric	2TIR	2643	1
6680	CH933809.1	4	24764193	24440484	24442275	Chimeric	2TIR	1792	1
6680	CH933809.1	4	24764193	24520907	24522561	X	Longer_2TIR	1655	1
6680	CH933809.1	4	24764193	24538620	24540276	X	Longer_2TIR	1657	1

Scf_6680	Proportion	Region Start	Region End	*Galileo* Obs	*Galileo* Exp		
Telomeric (3)	10.00%	0	2476419	0	1.4	Chi square test	
Central (2)	80.00%	2476420	22287773	0	11.2	**P-value=**	**4.35961000006307E-028**
Centromeric (1)	10.00%	22287774	24764193	14	1.4		
				14	14		

Table S4. Intrachromosomal distribution of *Galileo* elements (continuation).
Chromosome 5

Scaffold	GenBank_acc	Chr_arm	Scaffold_length	Galileo_start	Galileo_end	Galileo_subfam	Galileo_type	Galileo_length	Region
6496	CH933808.1	5	26866924	15292514	15294879	E	Longer_2TIR	2366	Central
6496	CH933808.1	5	26866924	23195067	23197892	F	NC_DD	2826	Central
6496	CH933808.1	5	26866924	25846816	25848819	F	2TIR	2004	3

Region	Proportion	Region Start	Region End
Telomeric (1)	10.00%	0	2686692
Central	80.00%	2686693	24180231
Centromeric (2)	10.00%	24180232	26866924

No enough copies for a Chi square test

Chromosome 6

Scaffold	GenBank_acc	Chr_arm	Scaffold_length	Galileo_start	Galileo_end	Galileo_subfam	Galileo_type	Galileo_length	Region
6498	CH933813.1	6	3408170	950693	951185	X	2TIR	493	Central
6498	CH933813.1	6	3408170	953555	955919	E	Longer_2TIR	2365	Central
6498	CH933813.1	6	3408170	1730250	1731243	F	SOLO	994	Central
6498	CH933813.1	6	3408170	1818872	1820002	F	2TIR	1032	Central
6498	CH933813.1	6	3408170	1999631	2001782	E	2TIR	697	Central
6498	CH933813.1	6	3408170	2177013	2181005	C	Longer_2TIR	3119	Central
6498	CH933813.1	6	3408170	2253149	2269701	F	NC	4036	Central
6498	CH933813.1	6	3408170	2381827	2382890	F	SOLO	1064	Central
6498	CH933813.1	6	3408170	2386095	2392524	D	NC	5721	Central
6498	CH933813.1	6	3408170	2407995	2408421	E	SOLO	427	Central
6498	CH933813.1	6	3408170	2514476	2515268	F	SOLO	793	Central
6498	CH933813.1	6	3408170	2522128	2522920	F	SOLO	793	Central
6498	CH933813.1	6	3408170	2541172	2544793	X	NC_DD	2423	Central
6498	CH933813.1	6	3408170	2560957	2561558	D	SOLO	602	Central
6498	CH933813.1	6	3408170	2609857	2611989	E	SOLO	1149	Central

Table S4. Intrachromosomal distribution of *Galileo* elements (continuation).

Scaffold	GenBank_acc	Chr_arm	Scaffold_length	Galileo_start	Galileo_end	Galileo_subfam	Galileo_type	Galileo_length	Region
6498	CH933813.1	6	3408170	2786970	2787863	F	SOLO	894	Central
6498	CH933813.1	6	3408170	2836237	2836700	E	SOLO	464	Central
6498	CH933813.1	6	3408170	2903343	2904985	X	2TIR	1643	Central
6498	CH933813.1	6	3408170	2966893	2969965	F	2TIR	2137	Central
6498	CH933813.1	6	3408170	2993866	2995242	E	2TIR	1377	Central
6498	CH933813.1	6	3408170	3022490	3023060	F	SOLO	571	Central
6498	CH933813.1	6	3408170	3120041	3121220	E	SOLO	1180	2
6498	CH933813.1	6	3408170	3281538	3283440	F	2TIR	1903	2

Region	Proportion	Region Start	Region End	*Galileo* Obs	*Galileo* Exp		
Region 1	10.00%	0	340817	0	2.3	Chi square test	
Central Region	80.00%	340818	3067352	21	18.4	P-val=	0.2583962888
Region 2	10.00%	3067353	3408170	2	2.3		
				23	23		

Table S**5**. Nearest genes to *Galileo* copies.

Scaffold	Start	Type	Group	Gene	*Galileo* position	Distance	D. melanogaster orthologous gene	Molecular function	Biological process
6540	5750063	2TIR	F	Dmoj-GI24072	downstream	29	Unknown	Unknown	Unknown
6540	31163990	Longer_2TIR	E	Dmoj-GI10679	upstream	69	Unknown	Unknown	Unknown
6500	29395284	2TIR	X	Dmoj-GI18249	downstream	131	Dmel\CG2614	Methyl-transferase (InterProScan)	Metabolic process
6540	14510521	Longer_2TIR	E	Dmoj-tRNA:GI25221	upstream	144	tRNA	tRNA	tRNA
				Dmoj-tRNA:GI25222	downstream	153	tRNA	tRNA	tRNA
6540	6132112	2TIR	C	Dmoj-GI23502	downstream	147	Unknown	Unknown	Unknown
				Dmoj-GI23503	upstream	219	CSN5	NEDD8 activating enzyme activity	Biological regulation; neuron differentiation; system development; multicellular organism reproduction; macromolecule modification; cellular component organization or biogenesis; localization; gamete generation; anterior/posterior axis specification; sensory organ development; dorsal/ventral axis specification
6496	23195067	NC_DD	F	Dmoj-GI18468	upstream	148	Dmel\CG7922	Helicase activity	ATP-dependent RNA helicase activity
				Dmoj-GI18348	upstream	152	Dmel\CG9890	Zinc ion binding	Unknown
6496	25846816	2TIR	F	Dmoj-GI21310	downstream	371	Nop60B (Nucleolar protein at 60B)	Pseudouridylate synthase activity	Wing disc development; ribosome biogenesis; germ cell development; rRNA processing; pseudouridine synthesis

Table S5. Nearest genes to *Galileo* copies (continuation).

Scaffold	Start	Type	Group	Gene	*Galileo* position	Distance	D. melanogaster orthologous gene	Molecular function	Biological process
6540	33286261	2TIR	F	Dmoj-GI21981	upstream	165	Orc2	DNA-binding	Mitotic chromosome condensation; DNA-dependent DNA replication initiation; cell proliferation; eggshell chorion gene amplification; mitotic spindle organization; DNA replication; chromosome condensation
				Dmoj-GI10788	downstream	371	T-cp1	Unfolded protein binding	Mitotic spindle organization; phagocytosis, engulfment
6680	23825194	SOLO	F	Dmoj-GI13965	downstream	209	GNBP1 (Gram-negative bacteria binding protein 1)	Protein binding	Peptidoglycan binding; immune response; peptidoglycan catabolic process; defense response to Gram-positive bacterium
6498	2786970	SOLO	F	Dmoj-GI14139	upstream	445	Nmdyn-D6	Nucleoside diphosphate kinase activity	Nucleoside diphosphate phosphorylation; GTP biosynthetic process; CTP biosynthetic process; UTP biosynthetic process
6328	1650720	2TIR	F	Dmoj-GI16179	upstream	463	Dmel\CG4332	Unknown	Unknown
6540	1072704	SOLO	F	Dmoj-GI23814	upstream	486	Dmel\CG16899 // FoxP	Sequence-specific DNA binding transcription factor activity	Regulation of transcription, DNA-dependent
6540	31163990	Longer_2TIR	E	Dmoj-GI10680	downstream	426	His4r	DNA binding;	Chromatin assembly or disassembly

Table S**6**. Intronic *Galileo* copies

Scaffold	Start	End	Type	Group	Gene	*FB* gene name	Intron length	D. melanogaster orthologous
6473	11762829	11764731	2TIR	C	Dmoj\GI15819	*Fb*gn0138568	5920	Dmel\CG9572
6482	269026	270625	2TIR	Chimeric	Dmoj\GI14384	*FB*gn0137136	119822	Unknown
6482	362528	364455	Longer_2TIR	E	Dmoj\GI14384	*FB*gn0137136	119822	Unknown
6482	614003	617184	NC_DD	D	Dmoj\GI14397	*FB*gn0137149	16289	Unknown
6482	617185	621442	NC_DD	D	Dmoj\GI14397	*FB*gn0137149	16289	Unknown
6482	2579294	2581638	2TIR	F	Dmoj\GI14475	*FB*gn0137227	48340	Dmel\S6kII
6498	2407995	2408421	SOLO	E	Dmoj\GI14130	*FB*gn0136884	1478	Dmel\C12.2
6498	2903343	2904985	2TIR	X	Dmoj\GI14010	*FB*gn0136764	55397	Unknown
6498	2993866	2995242	2TIR	E	Dmoj\GI14008	*FB*gn0136762	172415	Dmel\CG32627//NnaD
6498	3022490	3023060	SOLO	F	Dmoj\GI14008	*FB*gn0136762	172415	Dmel\CG32627//NnaD
6498	3120041	3121220	SOLO	E	Dmoj\GI14008	*FB*gn0136762	172415	Dmel\CG32627//NnaD
6500	30733241	30734538	2TIR	Chimeric	Dmoj\GI18277	*FB*gn0141016	65803	Unknown
6500	31339017	31339980	SOLO	E	Dmoj\GI18740	*FB*gn0141479	15508	Dmel\CG5708
6500	31884435	31886401	2TIR	C	Dmoj\GI18594	*FB*gn0141333	9452	Dmel\Cdk5alpha
6500	31888888	31889062	SOLO	F	Dmoj\GI18594	*FB*gn0141333	9452	Dmel\Cdk5alpha
6500	31891331	31891606	SOLO	X	Dmoj\GI18594	*FB*gn0141333	9452	Dmel\Cdk5alpha
6540	694288	695126	SOLO	F	Dmoj\GI23792	*FB*gn0146517	69549	Unknown
6540	722695	723349	SOLO	X	Dmoj\GI23792	*FB*gn0146517	69549	Unknown
6541	835755	836619	SOLO	F	Dmoj\GI14178	*FB*gn0136931	11198	Dmel\Stim
6541	1042036	1043771	Longer_2TIR	E	Dmoj\GI14176	*FB*gn0136929	47317	Dmel\CG8578
6541	1249195	1251094	2TIR	F	Dmoj\GI14213	*Fb*gn0136966	8704	Unknown
6541	1511326	1513666	2TIR	F	Dmoj\GI14170	*FB*gn0136923	16452	Dmel\Ranbp16
6680	24283772	24285562	Longer_2TIR	X	Dmoj\GI11297	*FB*gn0134058	37777	Dmel\Pka-C3
